# Full-scattering-matrix deterministic phonon boltzmann transport simulation

**DOI:** 10.1038/s41598-026-62876-7

**Published:** 2026-07-21

**Authors:** Y. Sungtaek Ju

**Affiliations:** Department of Mechanical and Aerospace Engineering, UCLA, 420 Westwood Plaza, Los Angeles, CA 90095 U.S.A.

**Keywords:** Phonon transport, Boltzmann transport equation, Scattering matrix, Singular value decomposition, Materials science, Mathematics and computing, Nanoscience and technology, Physics

## Abstract

Solutions to the phonon Boltzmann transport equation under the relaxation-time approximation (RTA) are fundamentally limited in that they do not account for the off-diagonal elements of the scattering matrix, which encode intermode energy redistribution. We find that the phonon in-scattering matrix is globally incompressible, requiring nearly its full rank for any useful Frobenius accuracy. The incompressibility worsens as the Brillouin zone is refined. We show that, despite this difficulty, one can develop a computationally efficient 3D BTE solver incorporating the complete scattering matrix by leveraging our two structural discoveries: the non-equilibrium phonon distribution inhabits a remarkably low-dimensional subspace of mode space regardless of how many phonon modes exist, and the leading singular modes of the scattering operator align selectively with this transport-active subspace. Consequently, truncation incurs negligible transport error even under large norm error. The phonon streaming operator’s mode-diagonal character further motivates a hybrid architecture that exploits these two properties. When applied to nanoscale structures emulating a fin field-effect transistor, our BTE solver quantifies a geometry-independent multiplicative correction to the temperature rise predicted under RTA. Our theoretical work and resulting BTE solver help enable rigorous study of phonon transport and systematic design of devices and structures in the ballistic and quasi-ballistic phonon transport regime.

## Introduction

As semiconductor structures and devices continue to scale down, non-diffusive heat transport is becoming increasingly significant. Experimental evidence that Fourier’s law breaks down in sub-micron silicon even at room temperature was first provided by Ju and Goodson^1^, who measured the thermal conductivity of silicon-on-insulator (SOI) thin films and found suppression consistent with phonon mean free paths being comparable to film thickness. This size effect was further confirmed using silicon nanowires by Li et al.^2^. These and many follow-up studies established that ballistic transport governs heat conduction at device-relevant scales in many important semiconductors.

The phonon BTE^3^ is widely used to model phonon transport, which in steady state is

1 $$\begin{aligned} \textbf{v}_\lambda \cdot \nabla f_\lambda (\textbf{r}) = -\sum _{\lambda '} W_{\lambda \lambda '} f_{\lambda '}(\textbf{r}) + \dot{Q}_\lambda (\textbf{r}), \end{aligned}$$

where $f_\lambda$ is the phonon distribution function for mode $\lambda = (\textbf{q}, s)$, $\textbf{v}_\lambda$ is the group velocity, $\dot{Q}_\lambda$ is a volumetric heat source, and *W* is the scattering matrix encoding all three-phonon Normal and Umklapp processes, computable from interatomic force constants^4,5^ (the operator encoding every phonon–phonon energy-exchange rate; its diagonal gives the usual relaxation times, its off-diagonal the coupling between modes).

Three communities have made complementary progress on solving Eq. (1), but none has achieved the combination of full *W*, three-dimensional geometry, and deterministic finite-volume discretization required for quantitative analysis of devices and structures. Table 1 summarizes the prior work context.

The *ab initio transport community*^4–7^ generates the full *W* and has established that off-diagonal scattering measurably affects phonon transport^8^. These methods, however, are restricted to periodic bulk geometries and cannot treat finite-domain, source-driven boundary value problems.

The *device BTE community*^9–12^ (see also physics-informed neural network approaches^13,14^) has developed methods for solving the BTE in 2D/3D geometries with realistic phonon dispersions and boundary conditions. The vast majority of existing solvers employ the relaxation time approximation (RTA), replacing $W_{\lambda \lambda '}$ with $-\delta _{\lambda \lambda '}/\tau _\lambda$ and ignoring intermode scattering correlations. A previous study solved the BTE deterministically in full 3D real and reciprocal space with first-principles input using a dual-relaxation (Callaway) model^15^. It attained beyond-RTA accuracy at reduced cost by approximating the scattering operator with normal and resistive relaxation channels; in the present study we retain the complete scattering matrix *W*, which is what enables the operator-spectrum analysis (incompressibility, relaxon spectrum, low-rank solution manifold) developed in this work. A recent review explicitly identifies reconciling RTA-based ballistic and hydrodynamic formulations as an outstanding necessity for thermal management in nanodevices^16^.

The *beyond-RTA Monte Carlo community*^17–19^ demonstrated that full-*W* BTE in finite domains differs from RTA — with errors reaching 10–30% at moderate Knudsen numbers in 2D materials and graphene ribbons. Beyond-RTA transport in finite geometries has also been studied with deviational Monte Carlo solutions of the Peierls–Boltzmann equation combined with thermal-transport measurements, e.g. in thin graphite^20^.

At first glance, the computational challenge appears straightforward: the $O(N_\lambda ^2)$ cost of handling *W* suggests truncation based on SVD (singular value decomposition) as the enabling technology, paralleling successful low-rank approximations in quantum chemistry and radiative transfer. We show, however, that this approach fails fundamentally for phonon-phonon scattering. The in-scattering operator $W_{\textrm{in}}$ requires 87–91% of its full SVD rank for even 1% Frobenius accuracy, and the required fraction worsens as the Brillouin zone is refined.

Conventional tensor-train compression along the full 6D mode-space indices likewise fails: we implemented and validated a complete TT-AMEn (tensor train alternating minimal energy) solver, which proved orders of magnitude slower than dense sweeps because the streaming operator is diagonal in mode space, making tensor-train coupling an overhead without computational benefit.

We also resolve why, despite the incompressibility of the scattering matrix, SVD truncation can still yield accurate solutions to the BTE, and how a computationally efficient solver can be built. We show that the non-equilibrium BTE solution occupies a low-rank subspace of mode space (its essential content is captured with far fewer degrees of freedom than the number of phonon modes). In 1D cases, two basis vectors describe the entire anisotropic distribution regardless of phonon mode count or device length. The leading SVD modes of $W_{\textrm{in}}$ are transport-selective: they align with this rank-2 subspace with selectivity 60–385*×*, so that the almost 90% of the SVD spectrum that is discarded acts entirely within the equilibrium mode subspace and has no effect on transport observables. This is reminiscent of the two-fluid phonon transport model^21^ where phonons are grouped into the “propagating” mode or the “reservoir” mode. Similar observations are made in 3D cases.

The end result is the first deterministic 3D BTE solver incorporating the full scattering matrix. When applied to a FinFET (fin field-effect transistor)-like structure, it quantifies the full-*W* correction — converged, geometry-independent, and physically interpretable.

In plain terms, as transistors shrink, the simplest model of how lattice vibrations (phonons) carry heat — in which each vibration relaxes on its own fixed timescale — becomes inaccurate, because the vibrations exchange energy with one another in ways that matter at these scales. The solver developed here keeps that full exchange (the complete “scattering matrix”) while allowing simulation of a three-dimensional device. The practical finding is that including the full exchange raises the predicted peak temperature rise, and — perhaps surprisingly — this correction barely changes as the device is made longer or shorter. A tension runs through the problem: the operator encoding all this mode-to-mode coupling is mathematically “incompressible” (it cannot be reduced to a few effective modes), yet the temperature field it produces is simple and smooth. The resolution of that tension is what makes a fast, accurate 3D solver possible, and it organizes the results that follow.

**Table 1:** Scope of prior work. All binary assessments reflect the scope of the cited work as reported. The final column records whether the method solves the phonon BTE with the complete off-diagonal scattering matrix for a finite geometry — the combination this work achieves for the first time in three dimensions.

Method	Full *W*?	Phys. BCs?	3D Si?	Det. FVM?	Full *W*, finite geom.?
ShengBTE/Phoebe/relaxons^4–6^	*\checkmark*	*×* (periodic)	*×*	*\checkmark*	*×* (bulk only)
Chiloyan et al. (2021)^8^	*\checkmark*	*×* (unbounded)	*×*	*\checkmark*	*×* (unbounded)
BTE-Barna (2023)^19^	*\checkmark*	*\checkmark* (2D)	*×*	*×* (MC)	*×* (2D only)
Landon & Hadjiconstantinou (2014)^17^	*\checkmark*	*\checkmark* (2D)	*×*	*×* (MC)	*×* (2D only)
Li & Lee (2019)^18^	*\checkmark*	*\checkmark* (2D)	*×*	*×* (MC)	*×* (2D only)
Beardo et al. (2025)^16^	*×* (RTA)	*\checkmark*	*\checkmark*	*\checkmark* (FEM)	*×* (RTA only)
GiftBTE (2024)^11^	*×* (RTA)	*\checkmark*	*\checkmark*	*\checkmark*	*×* (RTA only)
JAX-BTE (2025)^12^	*×* (RTA)	*\checkmark*	*\checkmark*	*\checkmark*	*×* (RTA only)
Liu & Guo (2024), dual-relaxation^15^	*×* (2-channel)	*\checkmark*	*×* (graphite)	*\checkmark*	*×* (approx. *W*)
**This work**	*\checkmark*	*\checkmark*	*\checkmark*	*\checkmark*	*\checkmark*

Abbreviations: Phys. BCs = physical boundary conditions in a finite domain; Det. FVM = deterministic finite-volume method; MC = Monte Carlo; FEM = finite-element method.

## Results

This section is organized around a single question with practical consequences for device thermal modeling: *how can a phonon transport solver that keeps the full mode-to-mode scattering be made fast enough for a 3D device, and what does keeping the full scattering change?* We answer it in four steps. Part I shows that the scattering operator itself is essentially *incompressible* — it cannot be approximated by a few modes, so naive low-rank or tensor-train compression of the operator does not help. Part II shows that, despite this, the *solution* the operator produces is very low-dimensional: the non-equilibrium part of the phonon distribution lives on a manifold of rank two to four. Part III reconciles these two facts through “transport selectivity”: only a small, identifiable part of the operator acts on the transport-relevant subspace, so a solver can be cheap without sacrificing accuracy. Part IV validates the resulting solver against an exact 1D reference and applies it to a 3D FinFET-like structure, where keeping the full scattering raises the predicted peak temperature rise — a correction that, strikingly, is nearly independent of device length. Readers primarily interested in the device results may read Part IV first and refer back to Parts I–III for the structural justification.

### Part I: The scattering operator is incompressible

#### Scattering channel count and matrix density

We first count how many mode-to-mode scattering pathways the scattering operator obtained from the Stillinger-Weber (SW) model of silicon (hereafter SW-silicon) contains. The count grows as the square of the number of modes, meaning the scattering matrix is genuinely dense — nearly every mode is coupled to nearly every other — which is the first sign that the operator will resist compression.

The off-diagonal entry $[W_{\textrm{in}}]_{\lambda \lambda '}$ is nonzero when a third mode *λ ''* simultaneously satisfies crystal-momentum conservation ($\textbf{q} \pm \textbf{q}' = \textbf{q}'' + \textbf{G}$) and energy conservation ($\omega _\lambda \pm \omega _{\lambda '} = \omega _{\lambda ''}$) on the Brillouin zone grid. For an $N^3$ Monkhorst–Pack grid with $M_{a}\approx 6N^3$ thermally active modes, the number of valid scattering triplets per mode grows as $O(N^3)=O(M_{a})$, giving a total channel count proportional to $M_{a}^2$ — the defining signature of a dense matrix. Table 2 confirms this empirically: at *N = 9* ($M_{a}= 4371$), the scattering matrix builder enumerates 35.8 million processes. Fitting the channel count to $M_{a}^\alpha$ gives $\alpha = 1.946 \approx 2$.

**Table 2:** Scattering channel count confirms $O(M_{a}^2)$ density.

*N*	$M_{a}$	Coalescence + Decay	$M_{a}^2$	Ratio
3	159	56,638	25,281	2.24
5	747	1,026,726	558,009	1.84
7	2,055	7,833,593	4,223,025	1.86
9	4,371	35,840,591	19,105,641	1.88

The ratio exceeds 1 because each scattering event involves three modes, giving roughly 2 matrix entries per triplet^22^. At all *N*, over 99% of entries of $W_{\textrm{in}}$ exceed a relative threshold of $10^{-4}$ (Table 3), confirming that $W_{\textrm{in}}$ is fully dense at all BZ grid sizes studied.

#### SVD rank requirement: global incompressibility

We next ask whether that dense operator can nonetheless be approximated by a modest number of dominant patterns (a low-rank approximation). It cannot: reproducing the operator to 1% accuracy still requires nearly 90% of its full rank, and this fraction does not fall toward zero as the grid is refined. We call this property incompressibility.

We compute the full SVD of $W_{\textrm{in}}$ at *N = 3, 5, 7, 9* and measure the rank fraction $r(\varepsilon )/M_{a}$ — the minimum fraction of the full SVD rank needed to achieve Frobenius tolerance *\varepsilon*:

2 $$\begin{aligned} \frac{\Vert W_{\textrm{in}}- W_{\textrm{in}}^{(r)}\Vert _F}{\Vert W_{\textrm{in}}\Vert _F} \le \varepsilon . \end{aligned}$$

**Fig. 1:**
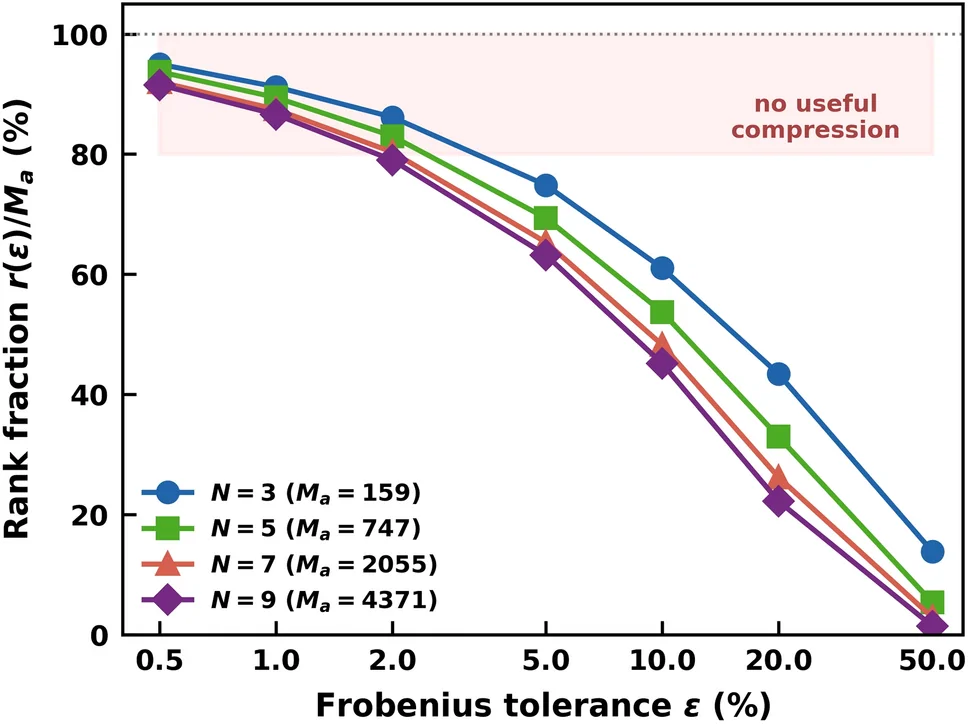
SVD incompressibility of $W_{\textrm{in}}$. Rank fraction $r(\varepsilon )/M_{a}$ required to achieve Frobenius tolerance *\varepsilon*, for *N = 3, 5, 7, 9*. The shaded region ($r/M_{a}> 80\%$) marks the regime with no useful compression. At $\varepsilon = 1\%$, the required fraction is 87–91% at all grid sizes.

The results are shown in Table 3 and Fig. 1. At $\varepsilon = 1\%$, the rank fraction is 91.2% (*N=3*), 89.4% (*N=5*), 87.4% (*N=7*), and 86.6% (*N=9*), respectively. The trend is a slow decrease: fitting $r(1\%)/M_{a}$ against $\log _{10}(M_{a})$ gives a slope of *-3.3%* per decade, extrapolating to 83% at the production ab initio grid size *N = 20* ($M_{a}\sim 50{,}000$). The fraction never approaches zero.

**Table 3:** SVD incompressibility and density of $W_{\textrm{in}}$.

*N*	$M_{a}$	*r(0.5%)*	*r(1%)*	*r(5%)*	*r(10%)*	$r(1\%)/M_{a}$	$\textrm{nnz}_{10^{-4}}/M_{a}^2$
3	159	151	145	119	97	91.2%	99.1%
5	747	700	668	518	401	89.4%	99.5%
7	2,055	1,891	1,797	1,342	991	87.4%	99.2%
9	4,371	4,001	3,785	2,762	1,972	86.6%	99.1%

**Fig. 2:**
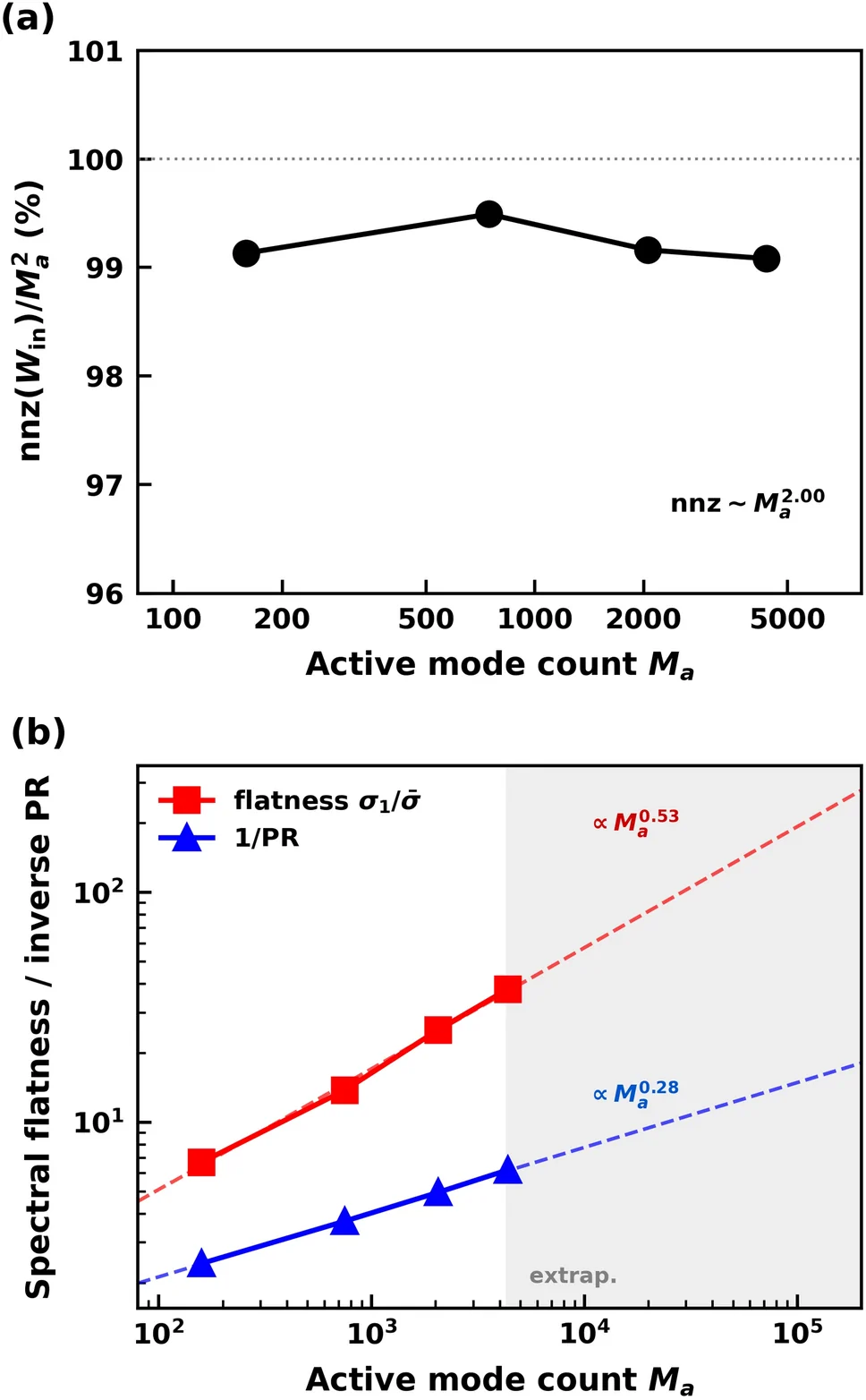
Spectral density and flatness scaling. (**a**) Non-zero entry fraction $\textrm{nnz}(W_{\textrm{in}})/M_{a}^2$ at threshold $10^{-4}$, showing *> 99%* density with power-law $\textrm{nnz} \propto M_{a}^{2.00}$ ($R^2 = 1.000$). (**b**) Spectral flatness $\sigma _1/\bar{\sigma }$ and $1/\textrm{PR}$ vs. $M_{a}$ on log–log axes, growing as $M_{a}^{0.53}$ and $M_{a}^{0.28}$ respectively.

The spectral flatness $\sigma _1/\bar{\sigma }$ (ratio of largest to RMS singular value) grows as $M_{a}^{0.53}$ — the spectrum becomes progressively more uniform as the BZ is refined (Fig. 2). The participation ratio $\textrm{PR} = (\sum _i \sigma _i)^2/(M_{a}\sum _i \sigma _i^2)$ decreases as $M_{a}^{-0.28}$, confirming no small subset of singular vectors captures a disproportionate share of the Frobenius norm. The Eckart–Young theorem^23^ guarantees these rank estimates are optimal. This shows that $W_{\textrm{in}}$ is globally incompressible via any low-rank representation at all physically relevant BZ grid sizes, and this incompressibility worsens with BZ refinement.

#### The relaxon spectrum is gapless

A natural way to compress a scattering operator is to diagonalize it and keep only the slowest collective modes (“relaxons”). This works only if there is a clear gap separating a few slow relaxons from the rest. We find no such gap: the spectrum is essentially gapless for the SW-silicon, so this route also fails to compress the operator.

An alternative compression route suggested by the relaxon theory^6,7^ (a relaxon being a collective eigenmode of the scattering operator, i.e. a combination of phonons that relaxes with a single timescale) is to diagonalize the symmetrized scattering matrix $\tilde{W} = C^{1/2}WC^{-1/2}$, where $C = \textrm{diag}(c_\lambda )$ is the heat-capacity matrix. If the eigenvalue spectrum had a gap between a small number of slow modes ($|\lambda _s| \ll |\lambda _{\max }|$) and the fast bulk, those slow modes would define a compressible transport subspace. Table 4 shows the gap ratio $|\lambda _1|/|\lambda _{M_{a}}|$: values are approximately 0.002–0.004 at all *N*, indicating an essentially gapless spectrum. The number of relaxons within 5% of $|\lambda _{\max }|$ grows as $M_{a}^{1.1}$ — approximately proportional to $M_{a}$ — providing no compression.

**Table 4:** Relaxon eigenvalue spectrum: gapless at all grid sizes.

*N*	$M_{a}$	Gap ratio $|\lambda _1|/|\lambda _{M_{a}}|$	Slow modes (1%)	Slow modes (5%)	Slow modes (10%)
3	159	0.00324	1	3	9
5	747	0.00315	2	2	18
7	2,055	0.00236	2	43	810
9	4,371	0.00409	2	55	1,552

#### Why tensor-train full-6D compression also fails: streaming diagonality

We also tried a tensor-train representation of the full six-dimensional problem (three mode/angle indices and three spatial indices). Although it compresses the data well, it is more than an order of magnitude slower than direct sweeps in practice, for a structural reason we now explain: the streaming part of the equation is already trivial mode-by-mode, so compressing it buys nothing.

A full six-dimensional TT-AMEn solver on the index set $(\lambda , \theta , \varphi , x, y, z)$ was implemented and validated: residual $6.6 \times 10^{-16}$ vs. the dense reference, 99.2% parametric compression, TT bond ranks 7–25 at the mode-space bipartitions (grid-independent). Despite these properties, production timing showed TT-AMEn to be more than an order of magnitude slower than dense mode-parallel sweeps at device scale.

The root cause is structural and general. The streaming operator $\textbf{v}_\lambda \cdot \nabla e_\lambda (\textbf{r})$ is diagonal in mode space: for each $(\lambda , \theta , \varphi )$, it reduces to a scalar advection equation on the spatial grid, solvable by a single upwind sweep in $O(N_{\textrm{spatial}})$. Dense sweeps exploit this directly. TT-AMEn couples all modes through the TT ranks at every site, paying $O(r^2 n_k r_A^2)$ per site per sweep even when no inter-mode coupling is required for streaming. The local operator formation (59% of sweep time) and dense local solve (37%) dominate, with the ratio TT/dense growing as the grid is refined. The TT-AMEn experiment establishes that the BTE solution lies on a ${\sim }25$-dimensional manifold in joint mode–spatial space (TT bond rank at the mode-spatial bipartition saturates at 25 regardless of truncation threshold for the *N = 5* run), a result we quantify in the next section.

### Part II: yet the solution is low-rank

In Part I we found the scattering operator to be incompressible: dense, full-rank to 1%, gapless, and resistant to tensor-train compression. This would seem to doom any low-cost solver. Part II shows why that conclusion is premature, by turning from the *operator* to the *solution* it produces.

#### The non-equilibrium distribution is low rank

The actual phonon distribution in a device under moderate heating is overwhelmingly near local-equilibrium (set by the local temperature); the transport physics lives in a small “deviation” on top of it. We show that this deviation, across slab thicknesses from ballistic to diffusive, is captured by only two independent patterns — it is rank two.

We first establish the rank-2 solution structure in the 1D slab geometry and then confirm the analogous result in 3D. The total phonon distribution $e_\lambda (x)$ is dominated by the local-equilibrium component $c_\lambda T(x)$ — a rank-1 field. The physically interesting part is the non-equilibrium deviation $\delta e_\lambda (x) \equiv e_\lambda (x) - c_\lambda T(x)$. We compute *δ e* at *N = 5* for converged full-*W* solves in a 1D slab at thicknesses *L = 20, 40, 100, 200, 500* nm. The non-equilibrium fraction $\Vert \delta e\Vert _F / \Vert e\Vert _F$ varies from 0.1% at *L = 20* nm to 0.02% at *L = 500* nm.

The SVD of the matrix $[\delta e]_{n,x}$ (shape *747 × 80*) yields the singular value structure shown in Table 5. The result is striking: *r(99%) = 2* at all slab thicknesses, from deeply ballistic to quasi-diffusive. The participation ratio $\textrm{PR} \approx 0.022$ means only 2% of the 747 modes carry 98% of the non-equilibrium variance.

**Table 5:** Solution manifold rank for the 1D slab geometry: non-equilibrium deviation *δ e = e - cT* and beyond-RTA correction $\Delta e = E_{\textrm{fW}} - E_{\textrm{RTA}}$ (*N=5*, $r_{\textrm{SVD}}=50$). All conductivities in $\mathrm{W\,m}^{-1} \textrm{K}^{-1}$. Kn is conductivity-weighted mean Knudsen number.

*L* (nm)	Kn	$r(99\%,\delta e)$	$\textrm{PR}(\delta e)$	$r(99\%,\Delta e)$
20	${\approx }\,7$	2	0.022	2
40	${\approx }\,3$	2	0.022	3
100	${\approx }\,1$	2	0.022	4
200	${\approx }\,0.5$	2	0.022	4
500	${\approx }\,0.2$	2	0.020	4

The physical origin of rank-2 is transparent in the 1D geometry. The BTE solution for a mode pair $(\lambda , \bar{\lambda })$ with $v_{\lambda ,x} = v$ and $v_{\bar{\lambda },x} = -v$ decomposes into symmetric and antisymmetric parts. The antisymmetric part $f^+ - f^-$ is the heat flux mode — it is proportional to $v_{\lambda ,x} \cdot (dT/dx)$ at leading order and constitutes singular vector 1. The symmetric correction from scattering constitutes singular vector 2. These two basis vectors span the entire non-equilibrium content regardless of how many phonon modes are present.

The column $r(99\%, \Delta e)$ in Table 5 shows the rank of the *beyond-RTA correction* $\Delta e = E_{\textrm{fW}} - E_{\textrm{RTA}}$. This rank stabilizes at 4 for *L ≥ 100* nm (the quasi-diffusive regime). That is, the beyond-RTA correction is 4-dimensional in mode space.

Turning now to the 3D FinFET-like geometry, we measure the mode-space rank of the non-equilibrium distribution directly via the TT-AMEn experiment. The TT bond rank at the mode-spatial bipartition — bond 2 (0-indexed) of the six-core TT chain $(\lambda , \theta , \varphi , x, y, z)$, which separates all mode and angular indices from all spatial indices — equals by definition the numerical rank of the matrix in Eq. (3):

3 $$\begin{aligned} \textbf{e}_{(\lambda \theta \varphi ),(xyz)} \in \mathbb {R}^{M_{a}N_\Omega \times N_{\textrm{cells}}}. \end{aligned}$$

For the *N = 5* run ($M_{a}= 747$, 128 angular directions), Supplementary Table S4 reports this bond rank as **25** for the full distribution *e*, saturating regardless of TT truncation threshold (Supplementary Section S6). The non-equilibrium deviation $\delta e = e - c_\lambda T(\textbf{r})$ has mode-space rank *≤ 24*. Both values are larger than those for the 1D slab geometry due to additional degrees of freedom available in 3D but are still more than an order of magnitude below $M_{a}= 747$ (*N = 5*) —and more than two orders of magnitude below the grid size $M_{a}= 13{,}179$ (*N = 13*)— confirming that the low-dimensional mode-space structure persists even in full 3D geometry with a volumetric line heat source, corner effects, and multi-directional temperature gradients.

### Part III: transport selectivity reconciles the two

The resolution is that the operator’s incompressibility and the solution’s low rank are not in conflict: they describe different subspaces. Most of the operator acts within the local-equilibrium subspace, which carries no heat flux; only a small part acts on the transport-relevant deviation. We make this precise with a *transport selectivity* measure.

The fact that the non-equilibrium distribution has a low rank has a profound implication. Despite the incompressibility of $W_{\textrm{in}}$ (requiring 89% of rank globally) discussed in Part I, a low-rank BTE solver can still provide accurate solutions. As an example, for the 1D slab, our rank-50 BTE solver achieved 0.77% error in the effective conductivity at *N* = 5 (Supplementary Table S3). The solution manifold has rank 4, and the leading 50 SVD modes of $W_{\textrm{in}}$ span this rank-4 subspace. The remaining almost 90% of the SVD spectrum, while large in Frobenius norm, acts entirely within the local-equilibrium subspace and has no effect on transport observables.

We quantify this through the transport selectivity

4 $$\begin{aligned} S = \frac{\Vert W_{\textrm{in}}- W_{\textrm{in}}^{(r)}\Vert _F / \Vert W_{\textrm{in}}\Vert _F}{|\Delta k^{(r)}| / k_{\textrm{ref}}}, \end{aligned}$$

where $\Delta k^{(r)} = k_{\textrm{ref}} - k^{(r)}$ is the conductivity error at rank *r* relative to the near-full-rank reference. *S ≫ 1* means the SVD truncation discards modes that are large in Frobenius norm but transport-inert. Table 6 shows results at all four BZ grid sizes.

**Table 6:** Transport selectivity *S* across BZ grid sizes and ranks for the 1D slab geometry (*L = 100* nm, $N_x = 80$). The transport observable is the effective thermal conductivity. All thermal conductivities in $\mathrm{W\,m}^{-1} \textrm{K}^{-1}$.

*N*	$M_{a}$	$k_{\textrm{RTA}}$	$k_{\textrm{dense}}$	$S(r{=}10)$	$S(r{=}30)$	$S(r{=}50)$	$S(r{=}100)$	$S(r{=}200)$
3	159	32.52	34.63	22.6	58.3	54.5	118.8	—
5	747	73.38	76.65	35.7	76.0	60.8	36.8	48.6
7	2,055	78.05	84.13	17.8	68.2	47.5	66.4	92.9
9	4,371	74.71	80.78	18.5	96.0	85.1	108.9	384.8

**Fig. 3:**
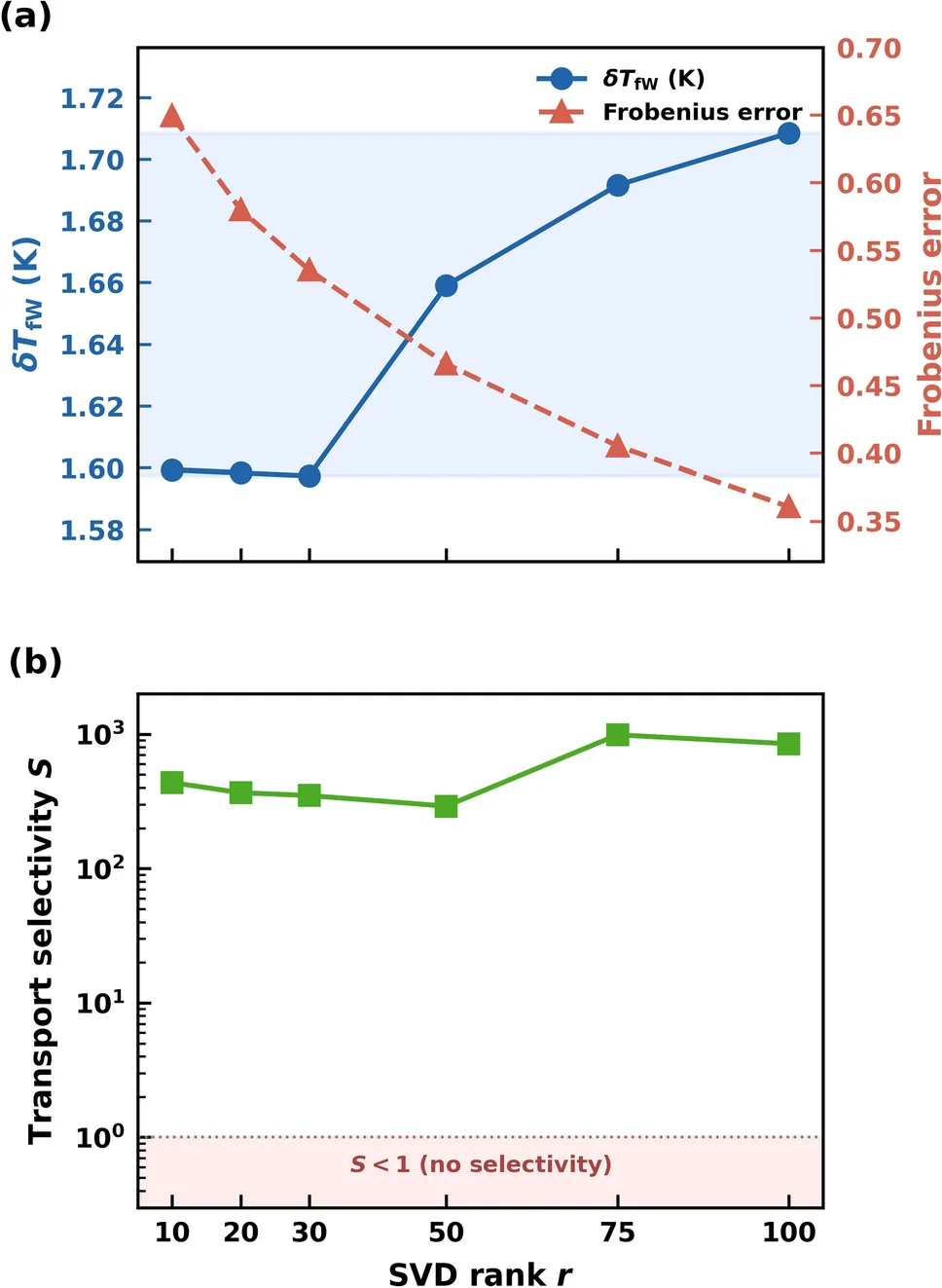
Transport selectivity of the SVD truncation (*N=5*, FinFET). (**a**) $\delta T_{\textrm{fW}}$ (blue, left axis) and Frobenius error (red, right axis) vs. SVD rank *r*. Blue band: 6.8% spread in *δ T*; Frobenius error varies 36–65%. (**b**) Transport selectivity $S = \varepsilon _F / (|\Delta k|/k_{\textrm{ref}})$ vs. *r* for the 3D FinFET at *N=5* (Table S6); values 291–989*×* throughout. Panel (**b**) is a property of the operator and the bulk conductivity and is independent of the device heat-source geometry; only the $\delta T_{\textrm{fW}}$ magnitude in panel (**a**) is geometry-specific.

Three observations follow from Table 6. First, *S ≫ 1* at all *N* and all tested ranks. Second, *S* grows with *N*: at *r = 200*, *S = 93* at *N=7* vs. *S = 385* at *N=9*. Third, *S* is non-monotone in *r*; the minimum transport error typically occurs in the range *r = 30*–100 depending on *N*. The rank *r = 50* used throughout this study is within this range for all *N*.

Figure 3 shows similar results for the 3D FinFET-like structure. The correction $\delta T_{\textrm{fW}}(N)$ is defined as the difference between the prediction under RTA and the prediction with the full scattering matrix: $T_{\textrm{RTA}}(N) - T_{\textrm{fW}}(N)$.

#### Why the streaming-diagonal structure is the enabling architecture

This subsection states the design principle that follows from Parts I–III — compress the scattering, sweep the streaming — and explains why it can lead to a fast yet accurate solver.

The streaming operator $\textbf{v}_\lambda \cdot \nabla$ is diagonal in mode space: for each mode *λ*, it acts on $e_\lambda (\textbf{r})$ independently without coupling to other modes. Our hybrid architecture — dense spatial sweeps for streaming + low-rank $W_{\textrm{in}}$ for scattering — exploits the streaming diagonality where it exists and the solution manifold compressibility where that exists.

This also explains why the TT-AMEn approach failed despite the solution lying on a 25-dimensional manifold: TT-AMEn applied low-rank structure to the streaming step (where mode-diagonality means rank 1 is exactly sufficient) rather than to the scattering step (where the rank-4 non-equilibrium structure provides genuine compression).

### Part IV: a validated solver and its device predictions

Parts I–III explain why a low-cost full-scattering solver is possible. Part IV demonstrates one. We verify it preserves equilibrium exactly, validate it against an analytic 1D reference, and then apply it to a 3D FinFET-like device (the simulated domain is shown schematically in Supplementary Section S8) to measure the quantity of practical interest: how much keeping the full scattering matrix changes the peak temperature, and how that change depends on device geometry.

#### Equilibrium invariant

With uniform boundary conditions at $T_0 = {300}\,\textrm{K}$ and no heat source, the solver reproduces the exact solution $e_\lambda (\textbf{r}) = 0$ to $\max _i |T_i - T_0| < 10^{-9}\,\textrm{K}$ at all BZ grid sizes.

#### 1D slab: analytic validation

Applying isothermal-diffuse wall conditions yields the per-mode effective conductivity ratio

5 $$\begin{aligned} \frac{k_{\textrm{eff},n}}{k_n^x} = \frac{1}{1 + 2\,\textrm{Kn}_n},\qquad k_n^x = \frac{c_n v_{n,x}^2\tau _n}{n_q V_{\textrm{uc}}},\qquad \textrm{Kn}_n = \frac{|v_{n,x}|\tau _n}{L}, \end{aligned}$$

and the analytic independent-mode-approximation (IMA) reference — in which each Brillouin-zone-sampled mode is suppressed independently by a Fuchs–Sondheimer factor evaluated with that mode’s projected velocity $v_{n,x}$ —

6 $$\begin{aligned} k_{\textrm{eff}}^{\textrm{Sond}} = \sum _n \frac{k_n^x}{1 + 2\,\textrm{Kn}_n}. \end{aligned}$$

For the SW-silicon, $\sum _n k_n^x = k_{\textrm{bulk}}= {148.0}\,\mathrm{W\,m}^{-1} \textrm{K}^{-1}$ (*< 0.003%* deviation by isotropy). Throughout, material-specific values (e.g. $k_{\textrm{bulk}}$, relaxation times, and the correction magnitudes below) are properties of this SW model and should not be read as quantitative predictions for crystalline silicon; the structural results (Parts I–III) are independent of this choice.

**Fig. 4:**
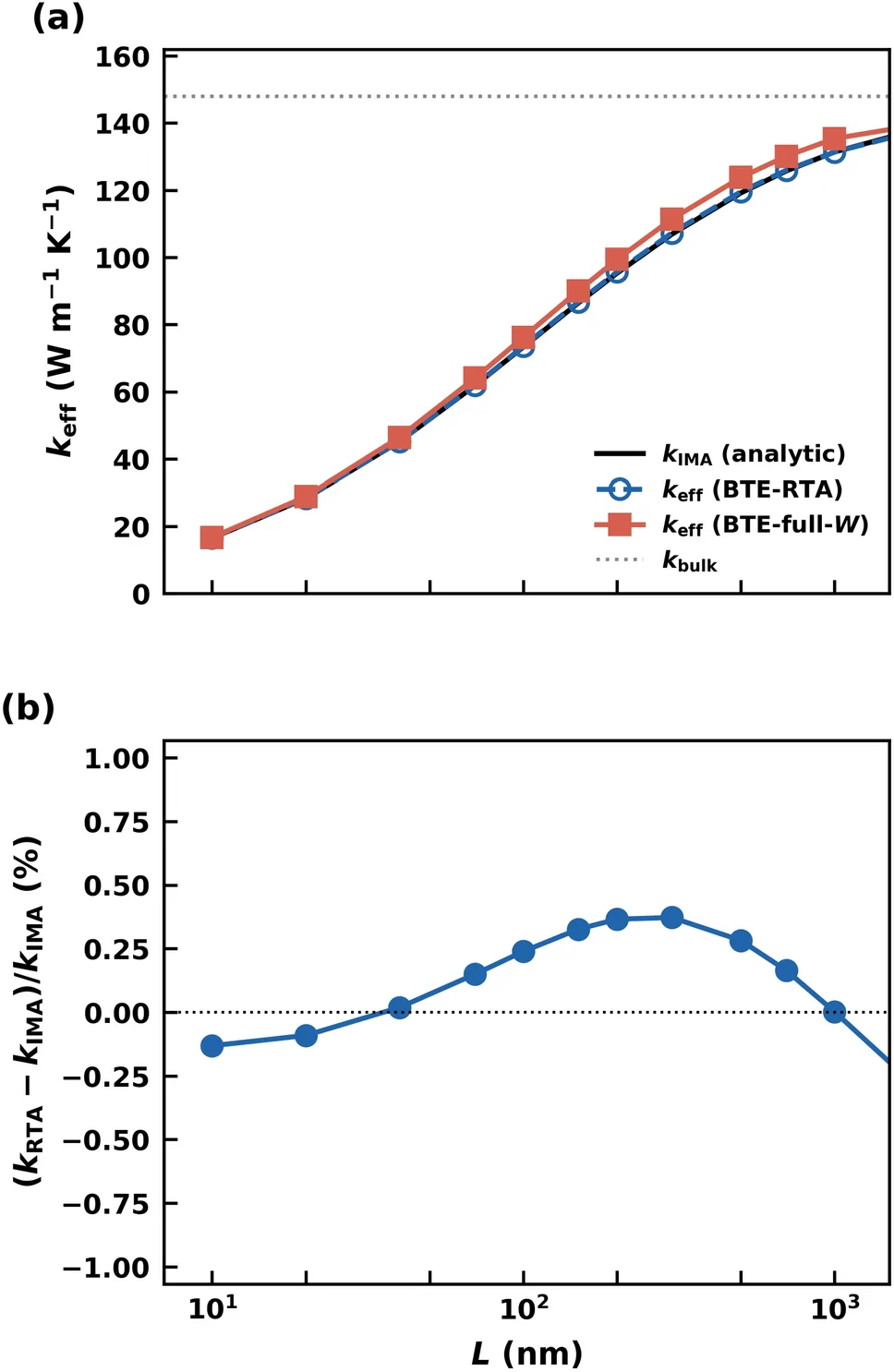
Validation: 1D slab BTE vs. exact analytic IMA reference. (**a**) Effective thermal conductivity $k_{\textrm{eff}}$ vs. slab thickness *L*, log-linear axes. Filled red squares: BTE full-*W* solver (SVD *r=50*). Open blue circles: BTE-RTA solver (*N=5*, $N_x=100$). Solid black line: analytic IMA reference Eq. (6). Dotted grey: $k_{\textrm{bulk}}= {148.0}\,\mathrm{W\,m}^{-1} \textrm{K}^{-1}$. (**b**) Relative deviation $(k_{\textrm{RTA}} - k^{\textrm{Sond}})/k^{\textrm{Sond}}$ vs. *L*. All values $\le 0.37\%$ over *L = 10*–1000 nm.

Table 7 and Fig. 4 compare $k_{\textrm{eff}}$ from the solver and Eq. (6) over *L = 10*–1000 nm. The maximum deviation between the RTA solver and the IMA is **0.37%** (at *L = 200*–300 nm). Under full *W*, $k_{\textrm{eff}}$ is 1.2–3.9% higher than the RTA across all converged *L*, consistent with the later results from the FinFET-like geometry.

Having established that the solver reproduces the exact 1D result to better than 0.4% and that the full-*W* correction there is 1–4%, we now turn to the 3D device, where the same correction is measured under realistic geometry and boundary conditions.

**Table 7:** 1D slab validation: BTE solver vs. analytic IMA (*N=5*, $N_x=100$). Values in $\mathrm{W\,m}^{-1} \textrm{K}^{-1}$. $\delta k_{\textrm{fW}}\% = (k_{\textrm{fW}} - k_{\textrm{RTA}})/k_{\textrm{RTA}}$.

*L* (nm)	$\textrm{Kn}_x$	$k^{\textrm{Sond}}$	$k_{\textrm{RTA}}$	err%	$k_{\textrm{fW}}$	$\delta k_{\textrm{fW}}$%
10	6.73	16.526	16.504	*-0.13*	16.704	*+1.21*
40	1.68	45.139	45.148	*+0.02*	46.418	*+2.81*
100	0.67	73.304	73.479	*+0.24*	76.171	*+3.66*
200	0.34	95.299	95.647	*+0.37*	99.382	*+3.90*
500	0.14	119.199	119.535	*+0.28*	123.803	*+3.57*
1000	0.07	131.387	131.390	0.00	135.392	*+3.05*

**SVD rank sensitivity.** $\delta T_{\textrm{fW}}$ varies by only 6.8% relative across ranks *r = 10*–100, while the Frobenius error varies from 65% to 36% (Fig. 3). The transport selectivity $S = \varepsilon _F/(|\Delta k|/k_{\textrm{ref}})$ is 291–989*×* across this range.

**DSA (Diffusion Synthetic Acceleration) fixed-point consistency.** With and without DSA, $T_{\max }$ agrees to $\le {0.5}\,\textrm{mK}$ at all BZ grid sizes.

#### 3D FinFET: BZ grid convergence

We check that the device prediction is numerically converged with respect to the reciprocal-space grid, and report the central result — the full-scattering correction to the peak temperature rise.

All computations use the FinFET-like geometry with $P = {10}\,\upmu \textrm{w}$ y-uniform line heat generation on the top 10 nm of the fin. Table 8 presents the peak fin temperature under RTA ($T_{\textrm{RTA}}$), under full-*W* ($T_{\textrm{fW}}$), the BZ-induced shift $\Delta T_{\textrm{BZ}}(N) = T_{\textrm{RTA}}(N) - T_{\textrm{RTA}}(5)$, and the correction $\delta T_{\textrm{fW}}(N) = T_{\textrm{RTA}}(N) - T_{\textrm{fW}}(N)$.

**Table 8:** BZ grid convergence of the full-*W* correction. Ratio $= \delta T_{\textrm{fW}}/ (T_{\textrm{RTA}}- 300)$. DSA-confirmed: $T_{\textrm{fW}}(N{=}13) = {311.154}\,\textrm{K}$, $T_{\textrm{fW}}(N{=}15) = {311.233}\,\textrm{K}$ ($\le {0.5}\,\textrm{mK}$ agreement).

*N*	*M*	$T_{\textrm{RTA}}$ (K)	$T_{\textrm{fW}}$ (K)	$\Delta T_{\textrm{BZ}}$ (K)	$\delta T_{\textrm{fW}}$ (K)	Ratio
5	747	315.761	314.102	0.000	1.659	10.53%
7	2,055	313.388	311.671	*-2.373*	1.717	12.82%
9	4,371	314.587	312.670	*-1.174*	1.917	13.14%
11	7,983	312.896	311.429	*-2.865*	1.467	11.38%
13	13,179	312.688	311.154	*-3.073*	1.534	12.09%
15	20,247	312.706	311.233	*-3.055*	1.473	11.59%

**Fig. 5:**
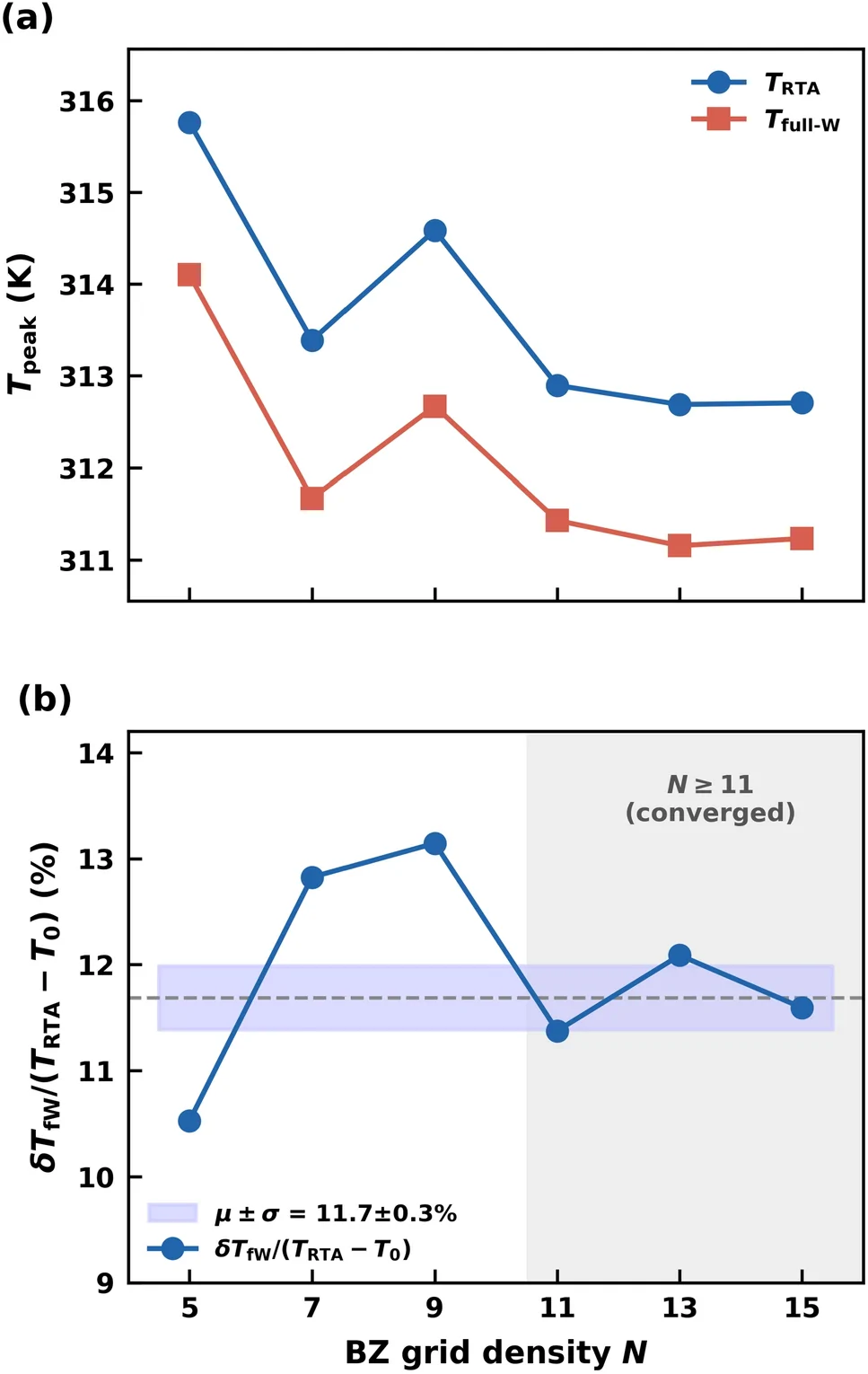
Brillouin zone grid convergence. (**a**) $T_{\textrm{RTA}}(N)$ (blue circles) and $T_{\textrm{fW}}(N)$ (red squares) vs. BZ grid density *N*. (**b**) Normalized correction $\delta T_{\textrm{fW}}/(T_{\textrm{RTA}}- T_0)$ vs. *N*; gray band marks the bounded band $11.7 \pm 0.3\%$ (*N ≥ 11*). The non-monotone behavior at *N = 7, 9* reflects the *Γ*-centered Monkhorst–Pack sampling artifact.

The absolute temperatures $T_{\textrm{RTA}}(N)$ exhibit non-monotone convergence at *N = 7, 9* — a known artifact of *Γ*-centerd Monkhorst–Pack sampling: at odd *N*, the grid does not close under $\textbf{q} \rightarrow -\textbf{q}$, producing disjoint sampling of long-MFP acoustic branches^24,25^. The correction $\delta T_{\textrm{fW}}(N)$ is the difference of two solutions on the *same* grid, and behaves qualitatively differently from the absolute temperatures: for *N ≥ 11* it oscillates within a bounded band with no monotone trend, rather than converging to a single value (Fig. 5). We therefore report it as a bounded, grid-robust correction. Taking *N = 11, 13, 15*:

7 $$\begin{aligned} \delta T_{\textrm{fW}}= 1.49 \pm 0.04\,\textrm{K} = 11.7 \pm 0.3\%\text { of the RTA temperature rise.} \end{aligned}$$

Two questions follow: does it depend on device geometry (Sec. 2.4), and where in the device does it act (Sec. 2.4)?

#### 3D FinFET: ballistic invariance

We vary the fin length over a decade and find the full-scattering correction (as a fraction of the temperature rise) barely changes — a device-design-relevant invariance.

**Fig. 6:**
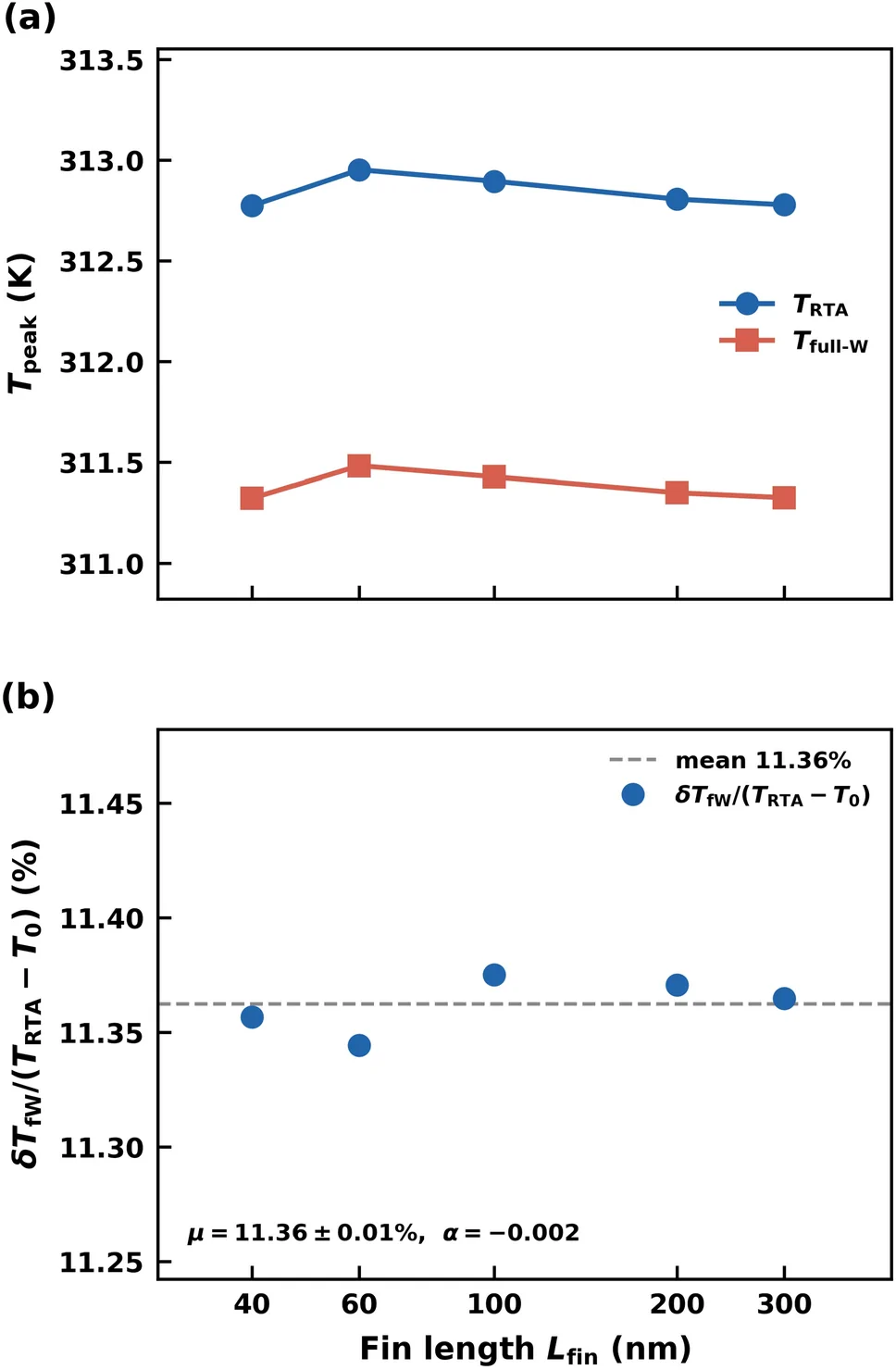
Ballistic invariance of the full-*W* correction. (**a**) Peak temperatures $T_{\textrm{RTA}}$ and $T_{\textrm{fW}}$ vs. $L_{\textrm{fin}}$. Both series are flat to within 0.2 K across a decade of device lengths. (**b**) Absolute correction $|\delta T_{\textrm{fW}}|$ vs. $L_{\textrm{fin}}$ with power-law fit (*α = -0.002*). Grey band: $\pm 1\sigma$. *N = 11.*.

Varying $L_{\textrm{fin}} = 40$–300 nm at fixed $P = {10}\,\upmu \textrm{w}$ and the converged grid *N = 11* gives the results shown in Table 9 and Fig. 6. A power-law fit gives $\alpha = -0.002 \approx 0$ (fin length explains a negligible fraction of the variance):

8 $$\begin{aligned} \frac{\delta T_{\textrm{fW}}}{T_\textrm{RTA}-T_0} = 11.36 \pm 0.01\%,\qquad \delta T_{\textrm{fW}}\propto L_{\textrm{fin}}^{\,\alpha },\ \ \alpha = -0.002. \end{aligned}$$

This ballistic invariance is a consequence of the low-rank solution structure: the non-equilibrium deviation *δ e* may be approximated as occupying a rank-2 subspace determined by the phonon dispersion and scattering physics, not by the device geometry. The value (11.36%, fixed *N=11*) is consistent with the BZ-convergence band ($11.7 \pm 0.3\%$, averaged over *N = 11, 13, 15*).

**Table 9:** Full-*W* correction vs. fin length (*N = 11*, line source).

$L_{\textrm{fin}}$ (nm)	$T_{\textrm{RTA}}$ (K)	$T_{\textrm{fW}}$ (K)	$|\delta T_{\textrm{fW}}|$ (K)	Ratio
40	312.774	311.323	1.451	11.36%
60	312.953	311.484	1.469	11.34%
100	312.896	311.429	1.467	11.38%
200	312.807	311.351	1.456	11.37%
300	312.779	311.326	1.452	11.36%

#### Spatial structure

**Fig. 7:**
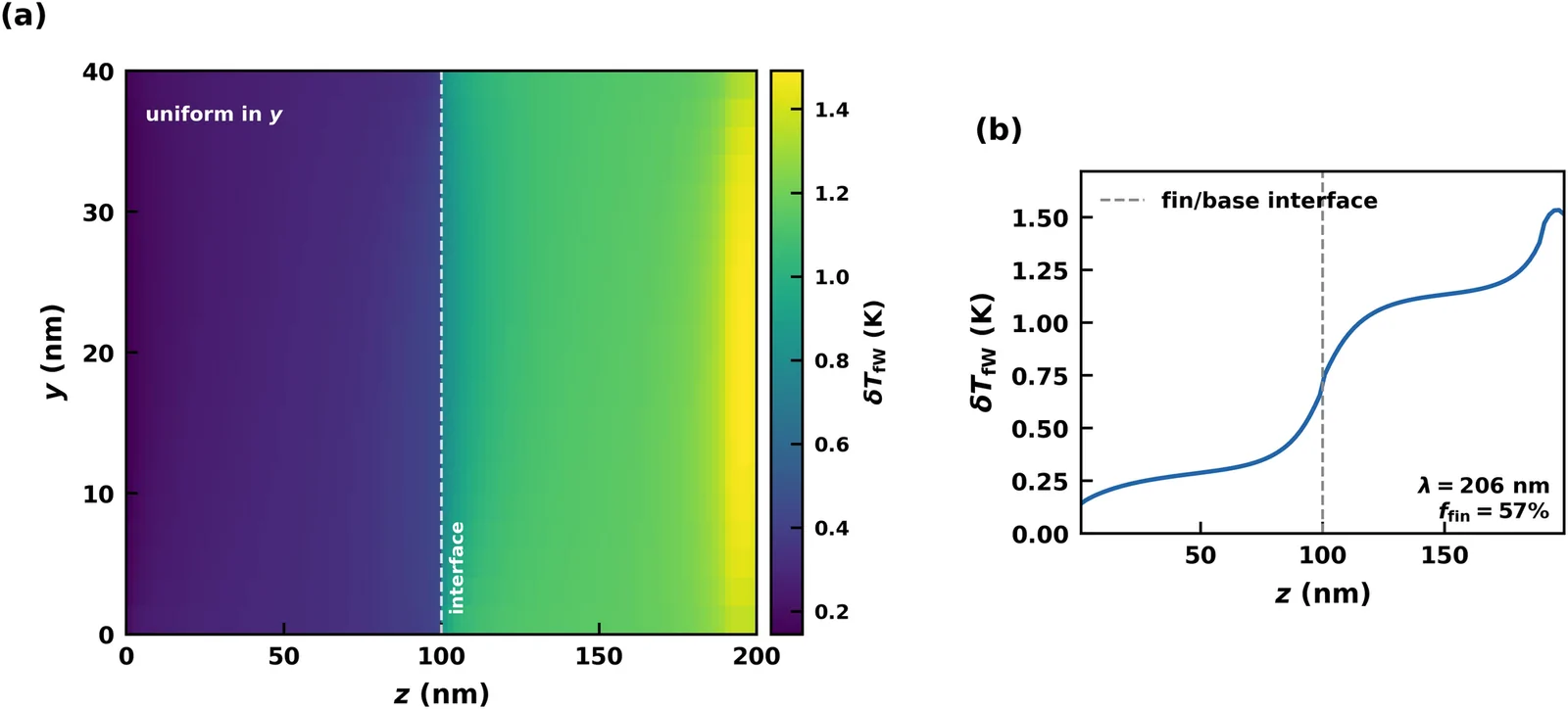
Spatial structure of the full-*W* correction field (*N=13*), line source. Peak $\delta T_{\max } \approx {1.53}\,\textrm{K}$ at the fin top; the field is uniform in *y*. Decay length *λ = 206* nm, $f_{\textrm{fin}} = 57\%$ retention. (**a**) 2D heatmap *δ T(y,z)* at the fin *x*-midplane, uniform in *y*. (**b**) Centerline *δ T(z)* vs. *z*; vertical dashed: fin–base interface; *λ = 206* nm, $f_{\textrm{fin}} = 57\%$ retention. *N = 13*.

We map where in the device the correction is largest and how far it spreads from the heat source. The correction field is uniform along the channel-depth direction *y* to within 1.4% in the fin interior (5.9% at the fin top, set by the diffuse end walls), confirming $\partial T/\partial y \approx 0$. The peak $\delta T_{\max } = {1.53}\,\textrm{K}$ occurs at the top of the fin; the correction decays toward the substrate with characteristic length *λ = 206* nm *= 2.1×* the fin height, with 57% retained in the fin and 43% in the base (Fig. 7).

## Discussion

### Physical interpretation of the correction

The full *W* introduces inter-mode energy redistribution: Normal processes transfer energy between branches without resistance, while Umklapp processes couple modes with different mean free paths, allowing long-MFP modes to scatter more efficiently into short-MFP modes. The net effect — for the SW phonon model — is an *∼*11% reduction in effective ballistic thermal resistance, uniform across device geometry. This 11% is specific to the SW potential; with ab initio force constants the magnitude may differ, but the qualitative physics and the structural conclusions are potential model-independent.

### The incompressibility result in broader context

Our result that $W_{\textrm{in}}$ requires 87–91% of full rank and worsens with refinement has direct implications for algorithms that attempt global low-rank compression of the phonon scattering operator. The relaxon picture^6,7^ achieves elegant results for bulk homogeneous systems; but the gapless spectrum we find ($|\lambda _1|/|\lambda _{M_{a}}| \approx 0.003$) confirms there is no natural truncation of the relaxon sum for the transport problem in finite devices. Green’s function approaches^8^ have so far been restricted to unbounded homogeneous media. The incompressibility result is a feature of three-phonon physics — the $O(N_q^6)$ channel count — not of the choice of potential or approximation level.

### Why the solution is nonetheless low-dimensional

Although $W_{\textrm{in}}$ is globally incompressible, the BTE solution is rank-2 in the non-equilibrium subspace. The BTE acts as a low-pass filter — fast-relaxing modes equilibrate locally and contribute only to the temperature, not to the heat flux. In 3D, the TT-AMEn mode-spatial bipartition measurement (Supplementary Section S6) confirms the analogous result empirically: the mode-space rank of the non-equilibrium deviation *δ e* is *≤ 24* for the FinFET-like geometry at *N = 5*, far below $M_{a}= 747$. The additional degrees of freedom relative to rank-2 reflect the 3D spatial boundary layers at all six walls and the fin–base interface, and the multiple independent heat-flux directions in the three-dimensional domain (see Supplementary Section S4 subsection ’Connection to TT-AMEn’).

### Choice of the Stillinger–Weber potential

The SW potential^26^ was chosen for three specific reasons. (i) Closed-form analytic expressions for FC2 and FC3 on any supercell enable exact verification of energy conservation $\sum _{\lambda '} W_{\lambda \lambda '} c_{\lambda '} = 0$ to machine precision. (ii) The relative scattering rates $W_{\lambda \lambda '}/|W_{\lambda \lambda }|$ are *α*-independent, where *α* is the fitted global timescale introduced to match the known bulk thermal conductivity of silicon. (iii) The SW potential is unambiguous and openly available.

We also note a regime boundary on the rank-2 result: in materials where Normal phonon–phonon scattering conserves crystal momentum strongly — the phonon-hydrodynamic regime, realized in graphene near room temperature and some III–V compounds at low temperature — a second slow hydrodynamic mode raises the mode-space manifold rank above 2.

The global incompressibility result is nevertheless unconditionally universal; only the rank-2 result is specific to the non-hydrodynamic regime, which still encompasses most device-relevant semiconductors at room temperature. Extension to DFT-derived force constants from ShengBTE^4^ or ALAMODE^27^, or machine-learned potentials^28^, is possible given our solver architecture.

### Why the structural results do not depend on the choice of potential

The three structural findings — $W_{\textrm{in}}$ incompressibility, rank-2 solution manifold, and transport selectivity — are consequences of the BTE’s mathematical structure, not of the specific phonon model. The $O(M_{a}^2)$ channel count and gapless relaxon spectrum are properties of three-phonon physics that hold for any material and any potential.

### Practical implications for full-*W* device BTE at production scale

For *N = 20* ($M_{a}\approx 50{,}000$): $W_{\textrm{in}}$ will require *∼*83% of full rank for 1% Frobenius accuracy. But the solution manifold will still be rank-2 to rank-4, and the transport selectivity will exceed *S> 1000×*. A rank *r ≈ 50*–100 SVD of $W_{\textrm{in}}$ will therefore achieve *< 1%* transport error at production BZ grids.

### On tensor-train methods

The full 6D TT-AMEn experiment established an empirical rank of *∼*25 for the BTE solution manifold in joint mode–spatial space (Supplementary Section S6). TT-AMEn is not the right vehicle for exploiting this compression because the streaming operator is mode-diagonal. The correct architecture compresses only $W_{\textrm{in}}$, leaving streaming as dense mode-parallel sweeps.

### Relation to coupled electro-thermal device simulation

Self-heating in aggressively scaled multi-gate transistors couples carrier transport to lattice heat generation, and conjoined electron–thermal studies have quantified the resulting thermal degradation during normal device operation^29^. Such studies typically close the phonon side with a relaxation-time or Fourier model. Our deterministic full-*W* solver supplies precisely this phonon-transport side beyond the relaxation-time closure: the natural coupling is a mode-resolved electron–phonon heat source $\dot{Q}_\lambda (\textbf{r})$ on the right-hand side of Eq. (1), through which the spatially and spectrally resolved Joule heating from a device electron solver feeds directly into full-scattering-matrix phonon transport. This is the forward path toward self-consistent electro-thermal simulation of FinFET, gate-all-around, and nanoribbon devices.

## Methods

### Governing equation

We solve the linearized steady-state BTE in deviation form, writing $e_\lambda (\textbf{r}) = \hbar \omega _\lambda [f_\lambda (\textbf{r}) - f_\lambda ^{\textrm{eq}}(T_0)]$ for a small deviation from reference equilibrium at $T_0 = {300}\,\textrm{K}$:

9 $$\begin{aligned} \textbf{v}_\lambda \cdot \nabla e_\lambda (\textbf{r}) = -\sum _{\lambda '} W_{\lambda \lambda '} e_{\lambda '}(\textbf{r}) + \dot{Q}_\lambda (\textbf{r}), \end{aligned}$$

with energy conservation $\sum _\lambda W_{\lambda \lambda '} c_{\lambda '} = 0$ (enforced analytically) and $T(\textbf{r}) = T_0 + \sum _\lambda e_\lambda / C_{\textrm{tot}}$.

### Phonon model

SW force constants^26^ on a $3\times 3\times 3$ supercell; three-phonon matrix elements from Fermi’s golden rule with Gaussian broadening $\sigma = {0.8}\,\textrm{THz}$. A single global timescale is fitted to obtain $k_{\textrm{bulk}}= {148.0}\,\mathrm{W\,m}^{-1} \textrm{K}^{-1}$. The raw stored *W* is converted to physical units ($\textrm{s}^{-1}$) via $W_{nn}^{\textrm{physical}} = -1/\tau _n$ (Supplementary Section S2). Resulting relaxation times span 1.86–327 ps. Energy conservation is enforced by a rank-1 null-space projection at operator application time. Because the SW model is used as a controlled testbed rather than a quantitative model of silicon, we refer to it as SW-silicon when reporting material-specific transport magnitudes. The SW description is known to misrepresent the optical-branch dispersion and group velocities; the device correction we report is therefore SW-specific in magnitude, while the structural results are potential-independent.

### Device geometry and heat source

FinFET-like structure: fin $20\times 40\times L_{\textrm{fin}}$ nm atop base $60\times 40\times 100$ nm. Isothermal substrate at 300 K; all side walls diffuse-adiabatic. Heat source (y-uniform line source): full $y \in [0, L_y]$, full *x*, $z \in [L_{\textrm{fin}}-10, L_{\textrm{fin}}]$ nm (top 10 nm of the fin); $\dot{Q} = 1.25 \times 10^{18} \mathrm{W\,m}^{-3}$ ($P = {10}\,\upmu \textrm{w}$). The source is uniform along the channel-depth direction *y*, so $\partial T/\partial y \approx 0$ in the fin; this de-activates the off-diagonal Cartesian conductivity arising from the SW group-velocity anisotropy (Supplementary Section S10). Full geometry specification is given in Supplementary Section S8.

### SVD compression of the scattering operator

The in-scattering operator $W_{\textrm{in}}= W + \textrm{diag}(1/\tau _\lambda )$ is compressed by truncated SVD at rank *r = 50*. Energy conservation is maintained after compression by a rank-1 correction at each matvec. The Frobenius error at *r = 50* is 47%; transport selectivity *S ≈ 48*–*85×* (Table 6) keeps transport error *< 1%*.

### Spatial discretization, angular quadrature, and iteration

Structured FVM is implemented on a $10\times 20\times 50$ fin mesh plus $30\times 20\times 50$ base mesh; $N_\Omega = 128$ angular directions. We adopt the upwind octant sweeps and Anderson mixing depth *m = 5* using temperature-space weights. Diffusion Synthetic Acceleration (DSA) reduces iteration counts by 15–20% with $\le {0.5}\,\textrm{mK}$ change in $T_{\max }$; derivation is in Supplementary Section S7. Convergence criterion is $\max _i|T_i^{(k+1)} - T_i^{(k)}|/\max _i|T_i^{(k)}| < 10^{-7}$. The full-*W* solver adds a scattering matvec to each iteration: $2rM_{a}N_{\textrm{cells}} \approx 5.97 \times 10^9$ FLOP, on top of the streaming cost $N_\Omega M_{a}N_{\textrm{cells}} \approx 7.65 \times 10^9$ FLOP shared with RTA, for a per-iteration ratio of $\approx 1.78\times$. The dominant driver of the measured overhead, however, is the larger iteration count required for convergence: the full-*W* solve takes 65–116% more iterations than RTA (Supplementary Table S8). On the compute-bound grids (*N ≤ 11*) the combination yields a measured total wall-time ratio of 1.7–*2.8×* over RTA (Supplementary Table S10). At *N = 13, 15* the ratio rises to 4–*5×* as the full-*W* working set approaches the 54 GB memory ceiling of the test machine and the solve becomes memory-bound rather than compute-bound.

### 1D slab analytic reference

The suppression function $(1 + 2\,\textrm{Kn}_n)^{-1}$ in Eq. (5) is exact for any single mode pair *(v,-v)* under RTA with diffuse boundary conditions.

### Solution manifold rank measurement

After a converged BTE solve, the SVD of the non-equilibrium deviation matrix $[\delta e]_{\lambda ,x} = [e]_{\lambda ,x} - c_\lambda T(x)$ gives the mode-space manifold rank at fractional variance threshold *p*: $r^* = \min \{r: \sum _{i=1}^r \sigma _i^2/\sum _i \sigma _i^2 \ge p\}$. Full data are in Supplementary Section S4.

## Supplementary Information

Supplementary Information.

## Data Availability

All relevant computed results are available in the main manuscript or supplementary information. The BTE solver code is available at https://doi.org/10.5061/dryad.dr7sqvbdq.

## Code availability

The solver source code is archived at the Dryad Digital Repository at https://doi.org/10.5061/dryad.dr7sqvbdq.
