# Supplementary material for: Full-scattering-matrix deterministic phonon boltzmann transport simulation
Source: Sci Rep. 2026 Jul 21;16:28485. doi: 10.1038/s41598-026-62876-7 (PMC13569850; doi:10.1038/s41598-026-62876-7)
Supplement: Supplementary file 1 [file 41598_2026_62876_MOESM1_ESM.pdf]

Supplementary Information

Full-Scattering-Matrix Deterministic Phonon Boltzmann Transport Simulation

Y. Sungtaek Ju[1*]

[1*]Department of Mechanical and Aerospace Engineering, UCLA, 420 Westwood Plaza, Los Angeles, CA, 90025, U.S.A..

Corresponding author(s). E-mail(s): sungtaek.ju@ucla.edu;

Contents

Sections

- Section S1: Complete validation chain (six tests)
- Section S2: Scattering matrix construction and unit conversion
- Section S3: SVD rank study methodology and extended data
- Section S4: Solution manifold rank: full data and physical derivation
- Section S5: Transport selectivity: full rank × N table
- Section S6: TT-AMEn implementation, validation, and failure analysis
- Section S7: DSA operator derivation
- Section S8: FinFET geometry specification
- Section S9: SW phonon calibration
- Section S10: Stillinger–Weber group-velocity anisotropy
- Section S11: Computational cost
- Section S12: Supplementary Tables

Tables (all collected in Section S12; cited from relevant sections)

- Table S1: Extended SVD rank fraction $r(\varepsilon)/M_a$ — cited in Section S3
- Table S2: Full solution manifold data — cited in Section S4
- Table S3: Complete transport selectivity data — cited in Section S5
- Table S4: TT-AMEn validation results, timing, and per-site profiling — cited in Section S6
- Table S5: FinFET geometry and computational parameters — cited in Section S8
- Table S6: SVD rank sensitivity (δT_{fW} vs. rank r) — cited in Section S1, test V6
- Table S7: Full BZ convergence with thermal resistance — cited in Section S1 test V5 and Section S7
- Table S8: Ballistic invariance full dataset with timing — cited in Section S8
- Table S9: Scattering channel counts vs. N — cited in Section S2
- Table S10: Computational cost — cited in Section S11

Section S1: Complete validation chain

Six independent tests confirm the solver pipeline.

V1 — Equilibrium invariant. With all walls at $T_0 = 300\,\mathrm{K}$ and no heat source, the exact solution is $e_\lambda(\mathbf{r}) = 0$, giving $T(\mathbf{r}) = T_0$. The solver reproduces this to $\max_i |T_i - T_0| < 10^{-9}\,\mathrm{K}$ for every BZ grid size $N \in \{5, 7, 9, 11, 13, 15\}$. This test covers all boundaries of the 1D slab and the

FinFET-like domain — including the fin apex, fin–base interface, and all side walls where Knudsen layers are strongest — and confirms the absence of projection-induced spatial artifacts throughout.

V2 — Bulk thermal conductivity. The calibrated relaxation times must reproduce $k_{\mathrm{bulk}} = 148.0\,\mathrm{W\,m^{-1}\,K^{-1}}$ via the kinetic formula $k = \frac{1}{3}\sum_n c_n v_n^2 \tau_n/(n_q V_{\mathrm{uc}})$, evaluated directly from the mode data without a BTE solve. Measured values satisfy $|\Delta k|/k_{\mathrm{bulk}} < 0.1\%$ for all $N \in \{3, 5, 7, 9\}$.

V3 — 1D slab, RTA vs. IMA. The upwind FVM must reproduce the IMA reference $k_{\mathrm{eff}}^{\mathrm{Sond}} = \sum_n k_n^x/(1 + 2\,\mathrm{Kn}_n)$ to within the inter-mode coupling residual. Maximum deviation 0.37% over $L =$ 10–1000 nm (Table 7 of main text).

V4 — Energy conservation. After SVD compression at rank r, the conservation null-vector condition $W^{(r)}\mathbf{c} = \mathbf{0}$ must hold to machine precision. The measured residual satisfies

$$\frac{\|W^{(r)}\mathbf{c}\|_\infty}{\max_{ij}|W_{ij}^{(r)}|} < 10^{-14} \tag{S1}$$

for all tested ranks $r =$ 10–100.

V5 — DSA fixed-point consistency. DSA must not alter the converged solution. With and without DSA, $T_{\max}$ agrees to $\leq 0.5\,\mathrm{mK}$ at $N = 13$ and $N = 15$. DSA reduces iteration counts by 15–20%. The complete BZ convergence data including thermal resistance values and iteration counts are given in Table S7.

V6 — SVD rank stability. δT_{fW} must be insensitive to SVD rank r once r is large enough to capture the transport-active subspace. The complete data are given in Table S6. The range of δT_{fW} across $r =$ 10–100 is 6.8% relative: the low ranks ($r =$ 10–30) slightly underestimate the correction, which plateaus by the production rank $r = 50$. Across this range the transport selectivity $S = \varepsilon_F/(|\Delta k|/k_{\mathrm{ref}})$ is 291–989× — the Frobenius error falls by nearly 30 percentage points (65% to 36%) while the bulk conductivity error stays below 0.16%. The selectivity here is a property of the operator and the bulk conductivity, and (unlike the δT_{fW} magnitude) does not depend on the device heat-source geometry.

Section S2: Scattering matrix construction and unit conversion

Three-phonon matrix elements

The scattering matrix $W_{\lambda\lambda'}$ is built from Fermi's golden rule applied to three-phonon processes on a Γ-centered Monkhorst–Pack grid of size N^3:

$$W_{\lambda\lambda'} = \frac{\pi}{4\hbar}\sum_{\lambda''}\left[|\mathcal{V}^{(3)}_{\lambda\lambda'\lambda''}|^2 \cdot g(\omega_\lambda, \omega_{\lambda'}, \omega_{\lambda''})\right]_{\mathrm{allowed}}, \tag{S2}$$

where $\mathcal{V}^{(3)}$ is the three-phonon coupling matrix element from the SW cubic force constants, g contains the Bose occupation factors for coalescence and decay processes, and "allowed" means both crystal momentum and energy conservation are satisfied within Gaussian broadening $\sigma = 0.8\,\mathrm{THz}$. Scattering channel counts at all four BZ grid sizes are given in Table S9, confirming the theoretical $O(M_a^2)$ scaling of the matrix density.

Unit conversion from stored to physical units

The critical property is that the ratio $|W_{nn}^{\mathrm{stored}}| \times \tau_n$ is constant (to machine precision) across all thermally active modes:

$$\frac{|W_{nn}^{\mathrm{stored}}|}{\mathrm{global\_ratio}} = \frac{1}{\tau_n}, \qquad \mathrm{global\_ratio} = \mathrm{median}\left\{|W_{nn}^{\mathrm{stored}}| \times \tau_n\right\}. \tag{S3}$$

Physical units are recovered by dividing W^{stored} by global_ratio. At $N = 3$: global_ratio $= 2.44\times10^{-10}$; at $N = 5$: global_ratio $= 7.83 \times 10^{-12}$.

This rescaling preserves: (i) detailed balance $c_n W_{nm} = c_m W_{mn}$; (ii) energy conservation $\mathbf{c}^\top W = \mathbf{0}$; (iii) the SVD singular value ratios. The incompressibility analysis in the main text is therefore correct regardless of whether stored or physical units are used.

Energy conservation projection

After unit conversion, energy conservation is enforced via an explicit rank-1 null-space projection applied at operator application time:

$$W \leftarrow W - \frac{(W\mathbf{c})\mathbf{c}^\top}{\|\mathbf{c}\|^2}. \tag{S4}$$

The residual $\|W\mathbf{c}\|_\infty / \max_i |c_i| < 10^{-14}$ after projection at all N and all tested ranks (test V4 in Section S1).

Spatial artifacts from the conservation projection. The correction term at each *matvec* has the form $\boldsymbol{\psi}\, s(\mathbf{r})$, where $\boldsymbol{\psi} = W_{\text{in}}^{(r)}\mathbf{c} \in \mathbb{R}^{M_a}$ is a fixed mode-space vector and $s(\mathbf{r}) = \mathbf{c}^\top \mathbf{e}(\mathbf{r})/\|\mathbf{c}\|^2 \propto T(\mathbf{r}) - T_0$. The correction adds the physically consistent structure of a local-equilibrium mode $\boldsymbol{\psi}$ modulated by the local temperature — precisely what is needed to cancel the spurious energy injection from the SVD-truncated scattering term.

The definitive validation is test V1: with isothermal walls at T_0 and no heat source, $s(\mathbf{r}) = 0$ identically, the correction term vanishes, and the solver reproduces $\max_i |T_i - T_0| < 10^{-9}$ K at all six boundaries. There are no projection-induced spatial artifacts.

Section S3: SVD rank study methodology and extended data

Methodology

For each BZ grid size N, the in-scattering matrix $W_{\text{in}} = W + \text{diag}(1/\tau_\lambda)$ is extracted on the thermally active mode subspace ($\omega > 0.1$ THz and $c_n > 10^{-30}$ J/K). The full SVD $W_{\text{in}} = U\Sigma V^\top$ is computed and the cumulative variance fraction

$$\text{cumvar}(r) = \frac{\sum_{i=1}^{r} \sigma_i^2}{\sum_{i=1}^{M_a} \sigma_i^2} \tag{S5}$$

is used to determine $r(\varepsilon) = \min\{r : \text{cumvar}(r) \geq 1 - \varepsilon^2\}$.

Extended rank fraction data

The complete rank fractions at tolerances $\varepsilon = 0.5\%$–20% are given in Table S1. At $\varepsilon = 1\%$, the rank fraction decreases slowly from 91.2% at $N = 3$ to 86.6% at $N = 9$, at a rate of -3.3% per decade of M_a. Extrapolating to the production ab initio grid ($N = 20$, $M_a \sim 50{,}000$) gives a projected fraction of $\sim 83\%$.

Spectral flatness diagnostics

Flatness ratio $\sigma_1/\bar{\sigma}$: values 6.68, 13.73, 25.17, 37.87 for $N = 3, 5, 7, 9$; power law $\sim M_a^{0.53}$. **Participation ratio** $\text{PR} = (\sum_i \sigma_i)^2/(M_a \sum_i \sigma_i^2)$: values 0.4125, 0.2703, 0.2025, 0.1617; power law $\sim M_a^{-0.28}$. **nnz fraction** at threshold 10^{-4}: $> 99\%$ at all N; scaling consistent with $\text{nnz} \sim M_a^{2.00}$ (see Table S9 for the underlying channel counts).

Section S4: Solution manifold rank — full data and physical derivation

Full data

The complete solution manifold rank data for the non-equilibrium deviation $\delta e = e - cT$ and for the beyond-RTA correction $\Delta e = E_{\text{fW}} - E_{\text{RTA}}$ at all tested 1D slab thicknesses are given in Table S2.

Physical derivation of rank-2

Consider the 1D slab BTE in deviation form at steady state:

$$v_{n,x}\frac{\partial e_n}{\partial x} = -\gamma_n e_n + [W_{\text{in}}\mathbf{e}]_n + \text{source}, \qquad n = 1, \ldots, M_a. \tag{S6}$$

The solution decomposes as $e_n(x) = c_n T(x) + \delta e_n(x)$.

Rank-1 contribution: at leading order, $[\delta e]_{n,x} = f_n \cdot g(x)$ is an outer product (rank-1), where $g(x) = dT/dx$ and f encodes the mode-dependent kinetic response to the gradient.

Rank-2 contribution: the first correction from inter-mode scattering via W_{in} mixes the leading-order flux mode f with a different pattern h that reflects the mode-coupling structure of W_{in}. This adds a rank-1 correction to give rank-2 overall.

Higher ranks and boundary layers: near the isothermal walls, a Knudsen layer contributes additional rank at $r(99.9\%)$ and higher. Bulk transport is dominated by the rank-2 contribution.

Invariance with L: the rank-2 structure depends on phonon dispersion and scattering rates, not on L. Changing L rescales $g(x)$ but not the mode-space pattern (f_n, h_n). This is the mechanism behind the ballistic invariance of δT_{fW}.

Connection to TT-AMEn: 3D mode-space rank

The TT bond rank at bond 2 (0-indexed) of the six-core TT chain $(\lambda, \theta, \varphi, x, y, z)$ equals by definition the numerical rank of the matrix $[\mathbf{e}]_{(\lambda\theta\varphi),(xyz)}$ (TT unfolding rank). Table S4 (Part a) reports this bond rank as **25** for the $N = 5$ run on the 3D FinFET-like structure ($M_a = 747$, 128 angular directions), saturating regardless of TT truncation threshold. Subtracting the rank-1 local-equilibrium component gives mode-space rank ≤ 24 for δe.

Note on the earlier "rank-14" estimate. An earlier validation run used 3 phonon bands ($M_a = 159$, $N = 3$ grid) on a $6 \times 5 \times 10$ mesh; that run saturated at bond rank 14. The 5-band ($M_a = 747$, $N = 5$) result of 25 supersedes this estimate. Both 14 and 25 are far below the respective M_a, confirming the same physical conclusion.

The higher 3D rank relative to 1D rank-2 arises from: (i) 3D spatial structure; (ii) multiple thermal boundary layers (fin–base interface, source region, six walls); (iii) 128 angular propagation directions vs. the $\pm$ pair in 1D. Despite this, rank ≤ 24 (δe) is far below M_a at all BZ grid sizes studied.

Section S5: Transport selectivity — full rank × N table

Table S3 gives the complete transport selectivity data at all four BZ grid sizes $N \in \{3, 5, 7, 9\}$ and all tested SVD ranks. The transport selectivity is defined as $S = \varepsilon_F/(|\Delta k|/k_{\text{ref}})$, where $\varepsilon_F = \|W_{\text{in}} - W_{\text{in}}^{(r)}\|_F / \|W_{\text{in}}\|_F$. All calculations at $L = 100$ nm, $N_x = 80$.

At $r = 200$ (4.6% of $M_a = 4371$ at $N = 9$), $S = 384.8\times$. As N grows toward the production ab initio grid ($N \sim 20$, $M_a \sim 50{,}000$), S will increase substantially beyond 384.8.

Section S6: TT-AMEn implementation, validation, and failure analysis

Implementation

A full six-dimensional TT-AMEn solver was implemented on the index set $(\lambda, \theta, \varphi, x, y, z)$. The streaming operator factorizes as a sum of 6 rank-2 matrix product operators:

$$\mathcal{A}_{\text{stream}} = \sum_{d \in \{x,y,z\}} V_\lambda^{(d)} \otimes \cos/\sin(\theta) \otimes \cos/\sin(\varphi) \otimes I_{x'} \otimes I_{y'} \otimes D_z^{\pm}, \tag{S7}$$

where $D_z^{\pm}$ is the upwind difference operator for direction d.

Validation results, timing, and per-site profiling

Table S4 summarizes the complete TT-AMEn characterization in three parts: Part (a) gives the validation results and bond ranks confirming correctness of the implementation; Part (b) gives the wall-time comparison across three grid sizes; Part (c) gives the per-site operation breakdown explaining the source of the 30–65× overhead.

The root cause is the streaming diagonality theorem: $\mathbf{v}_\lambda \cdot \nabla$ is diagonal in mode space, so dense sweeps exploit this directly while TT-AMEn couples all modes through the TT ranks at every site, paying $O(r^2 n_k r_A^2)$ per site per sweep even when no inter-mode coupling is required for streaming. The bottleneck (Table S4, Part c) is local operator formation (58.9% of sweep time), which reaches a

1680×1680 local system at bond 3 (for the 24x12x48 grid) with operator rank 11 and solution rank 25.

What TT-AMEn establishes despite the inefficiency

The BTE solution for the 3D FinFET-like structure lies on a $\sim$25-dimensional manifold in joint mode–spatial space (bond-2 rank = 25; ≤ 24 for δe), independent of TT truncation threshold. The correct architecture extracts this low-dimensionality at the right step: dense spatial sweeps for streaming + low-rank W_{in} matvec for scattering (building on the rank-2 structure established analytically in the 1D geometry, as detailed in Section S4, subsection 'Connection to TT-AMEn').

Section S7: DSA operator derivation

Energy balance equation

Summing Eq. (9) of the main text over all modes and using energy conservation:

$$\nabla \cdot \mathbf{J}(\mathbf{r}) = \dot{Q}_{\text{tot}}(\mathbf{r}), \qquad \mathbf{J} = \sum_{\lambda} \mathbf{v}_{\lambda} e_{\lambda}. \tag{S8}$$

Under the diffusion approximation, $\mathbf{J} \approx -k_{\text{diff}} \nabla T$, giving the diffusion correction equation for the error field δT after each transport sweep:

$$-\nabla \cdot [k_{\text{diff}}(\mathbf{r}) \nabla \delta T] = R(\mathbf{r}), \tag{S9}$$

where R is the energy-balance residual from the last sweep.

DCT decomposition in y

The FinFET geometry is translation-invariant in y. Expanding $\delta T(x, y, z) = \sum_m \hat{T}_m(x, z) \cos(m\pi y / L_y)$ and inserting into Eq. (S9):

$$\left[-\partial_{xx} - \partial_{zz} + \left(\frac{m\pi}{L_y} \right)^2 \right] \hat{T}_m(x, z) = \hat{R}_m(x, z), \qquad m = 0, 1, \ldots, N_y - 1. \tag{S10}$$

This is a 2D Helmholtz equation solved by sparse direct factorization ($O((N_x N_z)^{3/2})$ per mode). Total DSA cost: N_y independent 2D solves. Measured benefit: 15–20% iteration count reduction; the converged solution agrees with the non-DSA result to ≤ 0.5 mK (test V5 in Section S1). The complete iteration counts and thermal resistance values for all N are given in Table S7.

Section S8: FinFET geometry specification

The full geometry and computational parameters are given in Table S5 and Figure S1. The complete ballistic invariance dataset for $L_{\text{fin}} = 40$–300 nm ($N = 11$, line source) — including per-run iteration counts and wall times, confirming $\delta T_{\text{fW}} / (T_{\text{RTA}} - T_0) = 11.36 \pm 0.01\%$ throughout — is given in Table S8.

Section S9: SW phonon calibration

The Stillinger–Weber (SW) potential for silicon[1] gives closed-form analytic expressions for FC2 and FC3 on any supercell. The FC2 is computed once for a $3 \times 3 \times 3$ supercell (54 atoms) and shared across all BZ grid sizes via the Fourier transform $D(\mathbf{q}) = \sum_R \Phi_{0R} e^{i\mathbf{q} \cdot \mathbf{R}}$; total FC2 overhead ≈ 65 s on a Mac Studio (M1 Ultra CPU with 64 GB unified memory).

Three-phonon matrix elements use Gaussian broadening $\sigma = 0.8$ THz. A single global timescale α is fitted to $k_{\text{bulk}} = 148.0\,\text{W}\,\text{m}^{-1}\,\text{K}^{-1}$. At $N = 5$, $\alpha = 7.83 \times 10^{-12}$ gives $k_{\text{bulk}} = 148.000\,\text{W}\,\text{m}^{-1}\,\text{K}^{-1}$ ($< 0.001\%$ error). The fitted α rescales all diagonal entries $W_{nn} \to W_{nn}/\alpha$ uniformly; the off-diagonal relative rates $W_{nm}/|W_{nn}|$ are α-independent.

Section S10: Stillinger–Weber group-velocity anisotropy and the line-source geometry

On a Γ-centered Monkhorst–Pack grid, the SW group velocities carry a fixed [111] ellipticity: the Cartesian conductivity tensor $\kappa_{ab} = \sum_\lambda c_\lambda v_{\lambda,a} v_{\lambda,b} \tau_\lambda/(n_q V_{\rm uc})$ has equal diagonal entries ($\kappa_{xx} = \kappa_{yy} = \kappa_{zz} = 148.0\,\mathrm{W\,m^{-1}\,K^{-1}}$ to 0.0004%) but non-negligible off-diagonal entries ($\kappa_{xy}/\kappa_{xx} \approx 0.56$). The tensor is therefore traceless-clean (the scalar bulk conductivity is exact), and the principal axis is locked to [111] independent of grid size, identifying this as a fixed property of the SW force constants on a Γ-centered grid rather than q-grid sampling noise. An ab initio calculation on a symmetry-reduced grid gives vanishing off-diagonal entries, confirming the SW off-diagonal as a model/grid artifact.

For a device observable the off-diagonal κ_{xy} can in principle inject spurious flux through the $\kappa_{xy}\,\partial_y T$ term. The y-uniform line source used here makes $\partial T/\partial y \approx 0$ (verified: the correction field varies $\leq 1.4\%$ along y in the fin interior, Sec. 1), so this term is geometrically inactive and the reported correction is free of the anisotropy artifact. We verified independently that the normalized correction $\delta T_{\rm fW}/(T_{\rm RTA} - T_0)$ changes by < 0.5 percentage points between the drain-localized and line-source geometries at $N = 5$, confirming the artifact does not materially affect the headline result in either geometry.

Section S11: Computational cost

All device runs were executed single-process on an Apple M-series workstation (NumPy backend, no GPU), instrumented to record wall-clock time and peak resident memory. Table S10 reports both for the RTA and full-W solves across the Brillouin-zone convergence series. Memory is dominated by the dense angular sweep over the spatial grid; the two largest grids ($N = 13, 15$) are memory-limited at the $\approx 54\,\mathrm{GB}$ working ceiling of the test machine, setting the practical single-node limit of the present implementation.

Section S12: Supplementary Tables

Tables S1–S10 follow.

References

[1] Stillinger, F. H. & Weber, T. A. Computer simulation of local order in condensed phases of silicon. *Phys. Rev. B* **31**, 5262–5271 (1985).

Table S1 Extended SVD rank fraction $r(\varepsilon)/M_a$ at all tolerances. Discussed in Section S3. Values are rank count followed by fraction of M_a in parentheses. The spectral flatness $\sigma_1/\bar{\sigma}$ scales as $M_a^{0.53}$ and the participation ratio PR scales as $M_a^{-0.28}$, confirming that the spectrum has no exploitable gap at any BZ grid size.

N	M_a	$r(0.5\%)$	$r(1\%)$	$r(2\%)$	$r(5\%)$	$r(10\%)$	$r(20\%)$
3	159	151 (95.0%)	145 (91.2%)	137 (86.2%)	119 (74.8%)	97 (61.0%)	69 (43.4%)
5	747	700 (93.7%)	668 (89.4%)	620 (83.0%)	518 (69.3%)	401 (53.7%)	246 (32.9%)
7	2,055	1,891 (92.0%)	1,797 (87.4%)	1,651 (80.3%)	1,342 (65.3%)	991 (48.2%)	538 (26.2%)
9	4,371	4,001 (91.5%)	3,785 (86.6%)	3,454 (79.0%)	2,762 (63.2%)	1,972 (45.1%)	970 (22.2%)

Spectral flatness $\sigma_1/\bar{\sigma}$: 6.68, 13.73, 25.17, 37.87 at $N = 3, 5, 7, 9$; scaling $\sim M_a^{0.53}$. Participation ratio PR: 0.4125, 0.2703, 0.2025, 0.1617; scaling $\sim M_a^{-0.28}$. nnz fraction at threshold 10^{-4}: $> 99\%$ at all N.

Table S2 Full solution manifold data. Discussed in Section S4. $N = 5$, $r_{\mathrm{SVD}} = 50$. $\delta e = e - cT$; $\Delta e = E_{\mathrm{fW}} - E_{\mathrm{RTA}}$. All conductivities in $\mathrm{W\,m^{-1}\,K^{-1}}$. Conductivity values use $N_x = 80$; main text Table 7 uses $N_x = 100$ (within expected $O(1/N_x^2)$ spatial discretization error). Kn is the conductivity-weighted mean Knudsen number. The mode-space rank $r(99\%, \delta e) = 2$ at all 1D slab thicknesses, and $r(99\%, \Delta e) = 4$ for $L \geq 100$ nm.

L (nm)	Kn	k_{RTA}	k_{fW}	$\mathrm{frac}_{\mathrm{ne}}(\%)$	$r(99\%, \delta e)$	$r(99.9\%, \delta e)$	$\mathrm{PR}(\delta e)$	$r(99\%, \Delta e)$	$r(99.9\%, \Delta e)$
20	≈ 7	28.267	28.828	0.096	2	3	0.022	2	4
40	≈ 3	45.105	46.370	0.076	2	4	0.022	3	5
100	≈ 1	73.378	76.059	0.049	2	4	0.022	4	5
200	≈ 0.5	95.485	99.207	0.032	2	4	0.022	4	5
500	≈ 0.2	119.298	123.547	0.016	2	4	0.020	4	5

Table S3 Complete transport selectivity data. Discussed in Section S5. $S = \varepsilon_F/(|\Delta k|/k_{\mathrm{ref}})$, $\varepsilon_F = \|W_{\mathrm{in}} - W_{\mathrm{in}}^{(r)}\|_F/\|W_{\mathrm{in}}\|_F$. All calculations at $L = 100$ nm, $N_x = 80$. Conductivities in $\mathrm{W\,m^{-1}\,K^{-1}}$.

N	r	r/M_a	$k^{(r)}$	ε_F	$\|\Delta k\|/k$ (%)	S
$N = 3$ ($M_a = 159$, $k_{\mathrm{dense}} = 34.630$)						
3	10	6.3%	33.659	0.633	2.805	22.6
3	20	12.6%	34.009	0.517	1.792	28.9
3	30	18.9%	34.376	0.428	0.733	58.3
3	50	31.4%	34.444	0.293	0.537	54.5
3	75	47.2%	34.543	0.173	0.251	68.9
3	100	62.9%	34.603	0.091	0.077	118.8
3	150	94.3%	34.630	0.005	0.001	510.5
$N = 5$ ($M_a = 747$, $k_{\mathrm{dense}} = 76.646$)						
5	10	1.3%	75.251	0.649	1.821	35.7
5	20	2.7%	75.515	0.580	1.476	39.3
5	30	4.0%	76.107	0.535	0.704	76.0
5	50	6.7%	76.059	0.466	0.766	60.8
5	75	10.0%	76.001	0.405	0.842	48.1
5	100	13.4%	75.895	0.361	0.980	36.8
5	150	20.1%	75.940	0.292	0.922	31.7
5	200	26.8%	76.268	0.240	0.493	48.6
$N = 7$ ($M_a = 2{,}055$, $k_{\mathrm{dense}} = 84.125$)						
7	10	0.5%	81.125	0.635	3.566	17.8
7	20	1.0%	82.860	0.586	1.504	39.0
7	30	1.5%	83.437	0.558	0.818	68.2
7	50	2.4%	83.215	0.514	1.082	47.5
7	75	3.6%	83.385	0.475	0.881	53.9
7	100	4.9%	83.564	0.443	0.667	66.4
7	150	7.3%	83.750	0.392	0.446	87.9
7	200	9.7%	83.806	0.352	0.379	92.9
$N = 9$ ($M_a = 4{,}371$, $k_{\mathrm{dense}} = 80.779$)						
9	10	0.2%	78.048	0.626	3.380	18.5
9	20	0.5%	79.675	0.580	1.367	42.4
9	30	0.7%	80.314	0.553	0.575	96.0
9	50	1.1%	80.289	0.516	0.606	85.1
9	75	1.7%	80.370	0.484	0.505	95.8
9	100	2.3%	80.439	0.458	0.421	108.9
9	150	3.4%	80.574	0.419	0.254	165.4
9	200	4.6%	80.697	0.389	0.101	384.8

Table S4 TT-AMEn solver for the 3D FinFET: (a) validation results and bond ranks; (b) wall-time comparison with dense sweeps across three grid sizes; (c) per-site operation breakdown explaining the performance deficit. $N = 5$, $M_a = 747$, 128 angular directions. The bond-2 rank of 25 in Part (a) equals the numerical rank of the matrix $[\mathbf{e}]_{(\lambda\theta\varphi),(xyz)}$ by the definition of TT bond rank as a tensor unfolding rank. The non-equilibrium deviation $\delta e = e - c_\lambda T(\mathbf{r})$ has mode-space rank ≤ 24 at bond 2 (subtracting one rank-1 local-equilibrium component). Parts(b) and (c) use smaller test geometries than production to characterize TT-AMEn scaling

Test	Result
AMEn residual vs. dense reference	6.6×10^{-16}
Parametric compression vs. dense DOF	99.2%
TT bond ranks (mode bipartitions, bonds 0–2)	7, 19–22, **25**
TT bond ranks (spatial bipartitions, bonds 3–4)	$\sim n_z$ (grows with grid)
k_{bulk} agreement	$< 0.1\%$

Grid ($N_x \times N_y \times N_z$)	Dense	TT-AMEn	Ratio
$6 \times 3 \times 12$	6 s	∼5 min	∼50×
$12 \times 6 \times 24$	50 s	∼25 min	∼30×
$24 \times 12 \times 48$	330 s	∼6 hrs	∼65×

Operation	% sweep time	Scaling
Form local operator	58.9%	$O(r_A^2 r_x^2 n^2)$
Local dense solve	36.7%	$O((r_x n)^3)$
SVD truncation	0.5%	$O(rn)$
Environment update	4.0%	$O(r_A r_x^2 n)$

Table S5 FinFET geometry and computational parameters. Discussed in Section S8.

Parameter	Value
Fin	
Width	20 nm
Depth	40 nm
Height (L_{fin})	40–300 nm (parametric) / 100 nm (N-convergence)
Base (substrate)	
Width	60 nm
Depth	40 nm
Height	100 nm
Heat source	
Location	y-uniform line source: full $y \in [0, L_y]$, $z \in [L_{\text{fin}} - 10, L_{\text{fin}}]$, full x
Volumetric rate	1.25×10^{18} W m^{-3}
Total power	$P = 10\,\mu$W
Boundary conditions	
Bottom substrate	Isothermal, $T_0 = 300$ K
All side walls	Diffuse-adiabatic
Computational mesh	
Fin cells	$10 \times 20 \times 50$
Base cells	$30 \times 20 \times 50$
Total cells	80,000
Angular directions	128
Iteration	
Convergence criterion	$\max \lvert T^{(k+1)} - T^{(k)} \rvert / \max \lvert T^{(k)} \rvert < 10^{-7}$
Anderson depth	5
DSA	DCT in y, N_y independent 2D sparse solves

Note on diffuse-adiabatic boundary conditions: rigorous specular reflection requires exact mode-mirror pairing $(\mathbf{q}, s) \leftrightarrow (-\mathbf{q}, s)$. For Γ-centered Monkhorst–Pack grids with odd N, the grid is not closed under $\mathbf{q} \to -\mathbf{q}$, so all side walls use diffuse-adiabatic (zero net heat flux, isotropic re-emission). The isothermal substrate is implemented correctly for all N because it is an absorbing boundary.

Table S6 SVD rank sensitivity: δT_{fW} vs. rank r. Discussed in Section S1 (test V6). FinFET, $N = 5$, line source, $P = 10\,\mu$W. Production rank $r = 50$ is highlighted in bold. Range across $r = 10$–100: 1.597–1.708 K (6.8% relative), while the Frobenius error ε_F falls from 65% to 36% — the signature of transport selectivity (a large change in operator norm produces only a small change in the transport observable). Conservation residual $\|W^{(r)}\mathbf{c}\|_\infty = 0$ (machine precision) at all ranks, confirming test V4.

r	ε_F	$k_{\text{bulk}}^{\text{SVD}}$	$\Delta k\%$	δT_{fW} (K)	$T_{\text{max,fW}}$ (K)	n_{iter}
10	0.649	147.779	−0.149	1.599	314.161	38
20	0.580	147.765	−0.158	1.598	314.162	49
30	0.535	147.773	−0.153	1.597	314.163	48
**50**	**0.466**	**147.763**	**−0.160**	**1.659**	**314.102**	**53**
75	0.405	147.939	−0.041	1.692	314.069	46
100	0.361	147.937	−0.042	1.708	314.052	37

Table S7 Full BZ convergence with thermal resistance. Discussed in Section S1 (test V5) and Section S7. $R_{\mathrm{th}} = (T_{\mathrm{peak}} - T_0)/P$, $P = 10\,\mu\mathrm{W}$, $T_0 = 300\,\mathrm{K}$. Asymptote ($N \geq 11$): $\delta T_{\mathrm{fW}} = 1.49 \pm 0.04$ K $= 11.7 \pm 0.3\%$. DSA-confirmed values at $N = 13, 15$ agree to $\leq 0.5\,\mathrm{mK}$ with non-DSA results.

N	M	T_{RTA} (K)	T_{fW} (K)	ΔT_{BZ} (K)	δT_{fW} (K)	$R_{\mathrm{th}}^{\mathrm{RTA}}$ (MK/W)	$R_{\mathrm{th}}^{\mathrm{fW}}$ (MK/W)	Ratio
5	747	315.761	314.102	0.000	1.659	1.576	1.410	10.53%
7	2,055	313.388	311.671	−2.373	1.717	1.339	1.167	12.82%
9	4,371	314.587	312.670	−1.174	1.917	1.459	1.267	13.14%
11	7,983	312.896	311.429	−2.865	1.467	1.290	1.143	11.38%
13	13,179	312.688	311.154	−3.073	1.534	1.269	1.115	12.09%
15	20,247	312.706	311.233	−3.055	1.473	1.271	1.123	11.59%

Table S8 Ballistic invariance full dataset. Discussed in Section S8. All runs: $N = 11$, $P = 10\,\mu\mathrm{W}$, line source. Power-law fit: $\delta T_{\mathrm{fW}} \propto L_{\mathrm{fin}}^{-0.002}$ (fin length explains a negligible fraction of variance in δT_{fW}). Iteration counts and wall times (t_{fW}) are from the $N = 11$ line-source re-run on a single Apple M-series workstation. The full-W/RTA wall-time ratio is ≈ 1.9–$2.4\times$ for $L_{\mathrm{fin}} \leq 100$ nm, rising at the longest fins ($3.1\times$ at $L = 200$ nm, $6.0\times$ at $L = 300$ nm) as the larger spatial grids approach the memory ceiling of the test machine.

L_{fin} (nm)	T_{RTA} (K)	T_{fW} (K)	$\lvert\delta T_{\mathrm{fW}}\rvert$ (K)	Ratio	$n_{\mathrm{iter,RTA}}$	$n_{\mathrm{iter,fW}}$	t_{fW} (s)
40	312.774	311.323	1.451	11.36%	19	41	258
60	312.953	311.484	1.469	11.34%	18	30	192
100	312.896	311.429	1.467	11.38%	26	43	326
200	312.807	311.351	1.456	11.37%	24	48	575
300	312.779	311.326	1.452	11.36%	24	47	1364

Table S9 Scattering channel counts vs. N. Discussed in Section S2. Power law: total channels $\sim M_a^{1.946}$. The measured exponent is slightly below 2.0 because Gaussian broadening ($\sigma = 0.8\,\mathrm{THz}$) selects a frequency-width-dependent subset of all momentum-conserving triples. Over 99% of entries of W_{in} exceed a relative threshold of 10^{-4} at all N, confirming full matrix density.

N	$N_q = N^3$	M_a	Coalescence	Decay	Total	Total/M_a^2
3	27	159	28,866	27,772	56,638	2.24
5	125	747	514,454	512,272	1,026,726	1.84
7	343	2,055	3,921,238	3,912,355	7,833,593	1.86
9	729	4,371	17,936,351	17,904,240	35,840,591	1.88

Table S10 Measured wall-clock time and peak memory (line-source geometry, $P = 10\,\mu\mathrm{W}$).

N	M_a	mode	wall (s)	peak mem (GB)
5	747	RTA	22	2.5
		full-W	43	4.3
7	2,055	RTA	30	9.5
		full-W	83	13.9
9	4,371	RTA	95	19.0
		full-W	167	22.0
11	7,983	RTA	188	26.2
		full-W	328	37.9
13	13,179	RTA	326	44.7
		full-W	1352	54.4
15	20,247	RTA	733	54.4
		full-W	3522	54.4

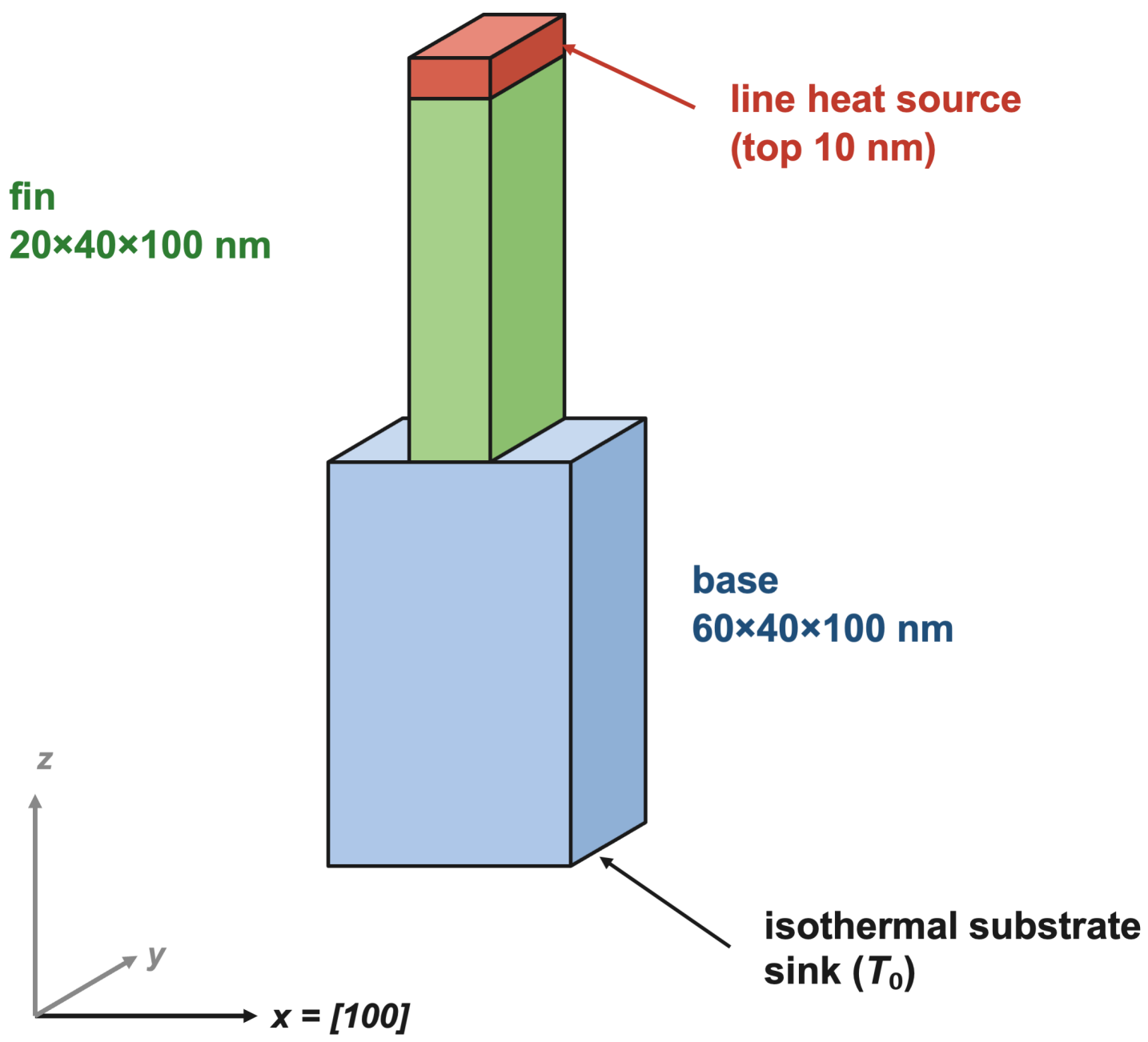

Fig. S1 Simulated FinFET-like domain. Fin (20 × 40 × 100 nm) on a substrate base (60 × 40 × 100 nm); isothermal substrate sink at T_0 at the base; diffuse-adiabatic side walls. The y-uniform line heat source occupies the top 10 nm of the fin across the full channel depth. Transport axis $x = [100]$.
